# Chondrosterins A–E, Triquinane-Type Sesquiterpenoids from Soft Coral-Associated Fungus *Chondrostereum* sp.

**DOI:** 10.3390/md10030627

**Published:** 2012-03-13

**Authors:** Hou-Jin Li, Ying-Lu Xie, Zhong-Liang Xie, Ying Chen, Chi-Keung Lam, Wen-Jian Lan

**Affiliations:** 1 School of Chemistry and Chemical Engineering, Sun Yat-sen University, Guangzhou 510275, China; Email: ceslhj@mail.sysu.edu.cn (H.-J.L.); 359160016@qq.com (Y.-L.X.); 540170598@qq.com (Y.C.); cklam@mail.sysu.edu.cn (C.-K.L.); 2 School of Pharmaceutical Sciences, Sun Yat-sen University, Guangzhou 510006, China; Email: 908129138@qq.com

**Keywords:** *Chondrostereum* sp., sesquiterpenoids, chondrosterins, marine fungus, cytotoxic activities

## Abstract

The marine fungus *Chondrostereum* sp. was collected from a soft coral *Sarcophyton tortuosum* from the South China Sea. This fungus was cultured in potato dextrose broth medium and the culture broth was extracted with EtOAc. Five new triquinane-type sesquiterpenoids, chondrosterins A–E (**1**–**5**), and the known sesquiterpenoid hirsutanol C (**6**), were isolated. The structures were elucidated mainly on the basis of NMR, MS, and X-ray single-crystal diffraction data. Chondrosterin A (**1**) showed significant cytotoxic activities against cancer lines A549, CNE2, and LoVo with IC_50_ values of 2.45, 4.95, and 5.47 μM, respectively.

## 1. Introduction

**Figure 1:**
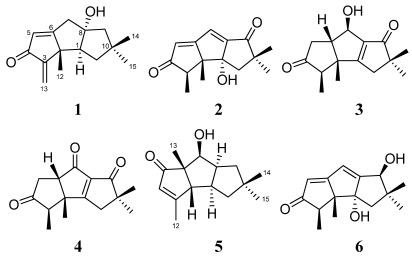
Chemical structures of compounds **1**–**6**.

*Sarcophyton tortuosum* is the most abundant soft coral in the shallow water regions of Hainan Sanya National Coral Reef Reserve, China. Our previous metabolites isolation work on this soft coral afforded six novel tetraterpenoids, methyl sartortuoate [1,2,3], methyl isosartortuoate [4], and methyl tortuoate A–D [5,6,7]. Our current studies concentrate on microorganisms, e.g., bacteria and fungi, associated with *Sarcophyton tortuosum*, with the main goal to discover novel metabolites with potent pharmacological properties. Forty-nine fungal strains were purified in our primary isolation. These fungi purifications were conducted using small-scale fermentation. The EtOAc extracts of their culture broth were screened for their cytotoxicity. The fungal strain, *Chondrostereum* sp. nov. (collection No. SF002), was cultured in glucose-peptone-yeast extract (GPY) medium and potato dextrose broth (PDB) medium. Both of the metabolites extracts showed significant cytotoxic activities with a 90% inhibitor ratio against the CNE2 cell line at 20 μg/mL. Investigation of the metabolites of *Chondrostereum* sp. cultured in GPY medium led to the isolation and structural determination of a new hirsutane sesquiterpenoid hirsutanol E, together with the known compounds hirsutanol A and gloeosteretriol. Hirsutanol A exhibited potent cytotoxic activities against various cancer cell lines, and can induce autophagic cell death by increasing ROS production [8,9]. The HPLC-MS data of GPY and PDB culture extracts showed distinct differences. This indicated that some metabolites in PDB medium were not present in GPY medium. Furthermore, the ^13^C NMR spectrum of the semi-pure EtOAc extract of *Chondrostereum* sp. in PDB medium showed many carbonyl carbon resonance signals (δ_C_ > 200). To the best of our knowledge, the carbonyl group is common in the naturally occurring hirsutane sesquiterpenoids. Therefore, the crude extract was assumed to be rich in hirsutane-type compounds, especially containing carbonyl groups. The isolation work on the extract from PDB medium resulted in the characterization of five new triquinane-type sesquiterpenoids, chondrosterins A–E (**1**–**5**), and the known sesquiterpenoid hirsutanol C (**6**). The structures were elucidated mainly based on the NMR, MS, and X-ray single-crystal diffraction experiments data. Chondrosterin A (**1**) showed significant cytotoxic activities. Herein we describe the structure elucidation and biological evaluation of these compounds (**1**–**6**, Figure 1).

## 2. Results and Discussion

**Figure 2:**
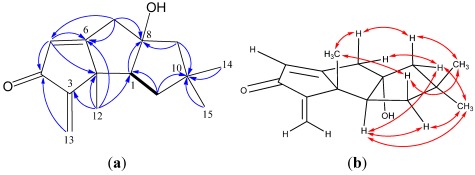
(**a**) ^1^H–^1^H COSY (bold line), main HMBC (arrow); and (**b**) selected key ROESY correlations of **1**.

Chondrosterin A (**1**) was obtained as yellowish oil. The molecular formula of **1** was established as C_15_H_20_O_2_, based on the HREIMS peak at *m/z* 232.1456 [M]^+^ and ^13^C NMR data (Table 1). The strong IR absorptions at 3433 and 1693 cm^−1^ indicated the presence of hydroxyl and conjugated carbonyl groups, respectively. The ^13^C NMR and DEPT spectra displayed three methyls, four methylenes, two methines and six quaternary carbons. One carbonyl carbon (δ_C_ 197.3), one trisubstituted double bond (δ_C_ 186.9; δ_C_ 125.0, δ_H_ 6.02, d, *J* = 1.5 Hz), and one terminal double bond (δ_C_ 154.0; δ_C_ 113.4, δ_H_ 5.89, s, and 5.16, s) represented three double bond equivalents. Thus, **1** must be tricyclic to account for the six double bond equivalents required by the molecular formula. The hydroxyl group was attached to quaternary carbon C-8 (δ_C_ 92.5), based on the large chemical shift. Two methyl groups with singlets at δ_H_ 1.12 and 1.19 were connected to quaternary carbon C-10 (δ_C_ 43.4), the other methyl group with singlet at δ_H_ 1.14 was connected to C-2 (δ_C_ 52.6), on the basis of their HMBC correlations (Figure 2). The cross-peaks of H-1/H-11 in ^1^H–^1^H COSY showed a partial structure –CHCH_2_– in this molecule. The HMBC correlations of H-5/C-4 and H-13/C-4 revealed a cross-conjugated dienone fragment. The HMBC correlations of H-1/C-8, H-5/C-2, H-7/C-5, H-7/C-6, H-7/C-8, H-9/C-8, H-9/C-10, H-11/C-10, H-12/C-1, H-12/C-2, H-12/C-3 and H-12/C-6 established the planar structure of compound **1**. To the best of our knowledge, the methyl group C-12 of natural hirsutane sesquiterpenoids always seems to be in β-orientation. The ROESY correlations of H-12/H-7β (δ_H_ 2.71), H-12/H-11β (δ_H_ 1.60) (Figure 2) established all these protons as β-oriented. In addition, ROESY correlations between H-1/H-9α (δ_H_ 1.90), H-1/H-11*α* (δ_H_ 1.76), H-1/H-15 allowed assignment of H-1 in α-orientation. 

**Table 1:** ^13^CNMR data of compounds **1**–**6**(125 MHz).

Position	1 ^a^	2 ^a^	3 ^a^	4 ^a^	5 ^a^	6 ^b^
1	60.6, CH	84.0, C	191.8, C	197.6, C	46.2, CH	82.1, C
2	52.6, C	62.9, C	53.1, C	49.5, C	63.8, CH	61.6, C
3	154.0, C	48.5, CH	53.1, CH	52.4, CH	182.6, C	47.0, CH
4	197.3, C	210.5, C	218.5, C	214.3, C	128.4, CH	210.4, C
5	125.0, CH	126.9, CH	34.4, CH_2_	36.4, CH_2_	211.1, C	116.8, CH
6	186.9, C	187.5, C	54.3, CH	59.5, CH	61.8, C	192.3, C
7	43.6, CH_2_	126.2, CH	67.4, CH	212.8, C	76.9, CH	116.0, CH
8	92.5, C	158.0, C	145.2, C	139.8, C	49.8, CH	173.9, C
9	55.9, CH_2_	207.6, C	209.1, C	202.6, C	39.9, CH_2_	75.8, CH
10	43.4, C	51.0, C	50.6, C	51.7, C	41.3, C	42.3, C
11	41.2, CH_2_	41.7, CH_2_	40.1, CH_2_	41.0, CH_2_	49.1, CH_2_	44.5, CH_2_
12	22.8, CH_3_	22.9, CH_3_	17.6, CH_3_	17.5, CH_3_	15.0, CH_3_	23.9, CH_3_
13	113.4, CH_2_	9.5, CH_3_	9.3, CH_3_	9.4, CH_3_	19.7, CH_3_	9.4, CH_3_
14	30.2, CH_3_	27.7, CH_3_	25.4, CH_3_	25.2, CH_3_	29.3, CH_3_	29.6, CH_3_
15	28.1, CH_3_	26.2, CH_3_	25.0, CH_3_	25.0, CH_3_	29. 2, CH_3_	23.0, CH_3_

^a^ Measured in CDCl_3_, and CDCl_3_ was used as an internal standard (δ_C_ 77.0); ^b^ Measured in DMSO-*d*_6_, and DMSO-*d*_6_ was used as an internal standard (δ_C_ 39.43).

**Figure 3:**
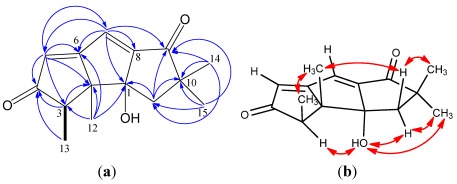
(**a**) ^1^H–^1^H COSY (bold line), main HMBC (arrow); and (**b**) selected key ROESY correlations of **2**.

Chondrosterin B (**2**) was isolated as yellowish oil. The HREIMS displays a molecular ion peak at *m/z* 246.1250 corresponding to the molecular formula C_15_H_18_O_3_. The UV λ_max_ 301 nm indicated the presence of a long conjugated system. Two carbonyl carbons (δ_C_ 210.5 and 207.6) and two trisubstituted double bonds (δ_C_ 126.9, δ_H_ 6.20, s, and δ_C_ 187.5; δ_C_ 126.2, δ_H_ 7.12, s, and δ_C_ 158.0) suggested that **2** also possessed a tricyclic system. Three methyl groups with singlets (δ_H_ 1.04, 1.18 and 1.40) and one methyl group with doublet (δ_H_ 1.16, d, *J* = 7.0 Hz) which connected with methine carbon C-3 (δ_C_ 48.5, δ_H_ 2.99, q, *J* = 7.0 Hz) are diagnostic resonance signals of hirsutane sesquiterpenoids. The hydroxyl group was placed at quaternary carbon C-1 (δ_C_ 84.0). The methylene at δ_C_ 41.7 was assigned to 11-position due to the HMBC correlations between H-11 and C-1, C-10, C-14, C-15. The HMBC correlations (Figure 3) of H-5/C-4, H-5/C-6, H-7/C-5, H-7/C-6, H-7/C-8, and H-7/C-9 allowed to establish the large conjugated system. 

The proton resonance signal of 1-OH showed a broad singlet at δ 1.94 in CDCl_3_ (500 MHz), whereas a sharp singlet signal at δ 5.52 in the solvent DMSO-*d*_6_ (400 MHz). ROESY data acquired in DMSO-*d*_6_ showed correlations of 1-OH/H-3, 1-OH/H-11 (δ_H_ 1.89), 1-OH/H-15, H-12/H-13, H-12/H-11 (δ_H_ 2.01), H-15/H-11 (δ_H_ 1.89), H-14/H-11 (δ_H_ 2.01). Based on these observations, 1-OH was assigned an α position, whereas H-12 (CH_3_) and H-13 (CH_3_) were assigned β positions. 

**Figure 4:**
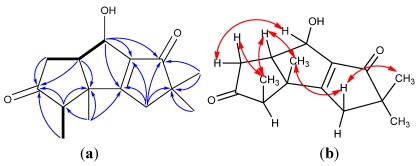
(**a**) ^1^H–^1^H COSY (bold line), main HMBC (arrow); and (**b**) selected key NOE correlations of **3**.

The molecular formula of chondrosterin C (**3**) was determined to be C_15_H_20_O_3_ due to its molecular ion peak at *m/z* 248.1405 [M]^+^ in the HREIMS spectrum. The IR spectrum displayed the characteristic absorptions of hydroxyl group (3355 cm^−1^), ketone carbonyl (1734 cm^−1^), and α,β-unsaturated carbonyl group (1684 cm^−1^). The ^13^C NMR data showed signals for one tetrasubstituted double bond (δ_C_ 191.8 and 145.2), two carbonyl groups (δ_C_ 218.5 and 209.1), and one tertiary carbon bearing one hydroxyl group (δ_C_ 67.4, C-7). The ^1^H NMR spectrum indicated the presence of three methyl singlets (δ_H_ 1.08, H-12; 1.17, H-14; and 1.16, H-15), and one methyl doublet (δ_H_ 1.05, d, *J* = 7.0 Hz, H-13). The ^1^H–^1^H COSY correlations of H-5/H-6, H-6/H-7 and H-3/H-13 revealed the presence of two fragments –CH_2_–CH–CHOH– and –CH–CH_3_ (Figure 4). Two carbonyl groups at δ_C_ 218.5 and 209.1 were placed at C-4 and C-9, respectively, based on the HMBC correlations of H-3/C-4, H-5/C-4, H-7/C-9, H-11/C-9, and H-14/C-9. The tetrasubstituted double bond was placed between C-1 and C-8, which was supported by the HMBC correlations of H-7/C-1, H-7/C-8, H-11/C-1, H-11/C-8, and H-12/C-1. By a combination of the ^1^H–^1^H COSY and HMBC spectra, the structure of **3 **could be established. A NOE experiment showed that selective irradiation at δ 1.05 (H-13) gave a clear signal enhancement of H-5 (δ 2.32) and H-6 (δ 2.83). Likewise, irradiation of the H-12 resonance at δ 1.08 gave NOE’s at the signals of H-5 (δ 2.32), H-6, and H-11 (δ 2.48). Irradiation of H-14 at δ 1.17 caused an NOE enhancement of H-11 (δ 2.48). Irradiation of the H-7 at δ 4.80 gave NOE’s to H-5 at δ 2.91. So, H-5 (δ 2.32), H-6, H-11 (δ 2.48), and two CH_3_ (H-12 and H-13) were determined as β-orientation, H-3 and H-7 were assigned as α-orientation.

**Figure 5:**
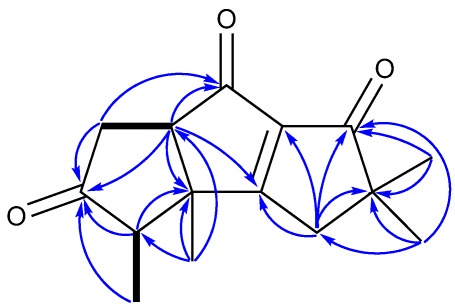
^1^H–^1^H COSY (bold line) and main HMBC (arrow)correlations of **4**.

**Figure 6:**
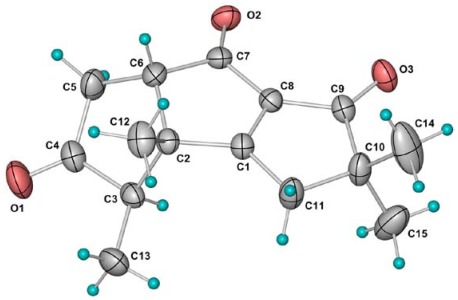
Molecular structure of **4 **in the crystal. Thermal ellipsoids are plotted at 30% probability level.

Chondrosterin D (**4**) was obtained as colorless crystals. The molecular formula was deduced as C_15_H_18_O_3_, based on the HREIMS, which showed a molecular ion peak at * m/z *246.1255 [M]^+^ (calculated for C_15_H_18_O_3_, 246.1250). The ^13^C NMR and DEPT spectra showed signals corresponding to four methyls, two methylenes, two methines, and seven quaternary carbons. Its NMR spectra showed the following functionalities: three carbonyl groups (δ_C_ 214.3, C-4; 212.8, C-7; 202.6, C-9), one tetrasubstituted double bond (δ_C_ 197.6, C-1; 139.8, C-8), four methyl groups, giving three singlets (δ_H_ 1.20, 1.25 and 1.28) and one doublet (δ_H_ 1.15, d, *J* = 7.0 Hz). Its IR spectra exhibited strong absorptions at 1737, 1687, and 1610 cm^−1^and supported the presence of the separate ketone and α,β-unsaturated carbonyl group. ^1^H–^1^H COSY indicated two partial structures, –CHCH_3_ and –CHCH_2_–, in this molecule (Figure 5). The planar structure of **4 **could be established, based on its HMBC correlations of H-3/C-2, H-3/C-4, H-5/C-4, H-6/C-1, H-6/C-2, H-6/C-7, H-11/C-1, H-11/C-8, H-11/C-9, H-11/C-10. Compound **4** is an unprecedented hirsutane sesquiterpenoid which bears three ketone carbonyl groups in the molecule. Finally, the structure and relative configuration of **4** was also confirmed by X-ray crystallography (Figure 6). The molecules related by the two-fold screw axis along the *b*-axis are joined by the weak C11–H…O1 hydrogen bonds to form a helical ribbon. Adjacent ribbons related by simple translation along the *a*-axis are further connected by pairs of weak C3–H…O2 hydrogen bonds to form a three-dimensional network.

**Figure 7:**
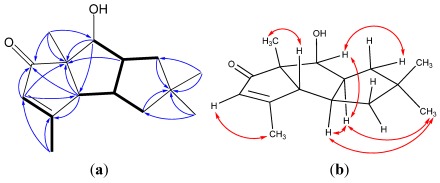
(**a**) ^1^H–^1^H COSY (bold line), main HMBC (arrow); and (**b**) selected key ROESY correlations of **5**.

Chondrosterin E (**5**) was obtained as white solid. The molecular formula of **5** was deduced as C_15_H_22_O_2_ by HREIMS and NMR data. This molecule contained the following diagnostic functional groups: one carbonyl carbon (δ_C_ 211.1), one trisubstituted double bond (δ_C_ 128.4, δ_H_ 5.73, q, *J* = 1.0 Hz; δ_C_ 182.6), three methyl groups with singlets (δ_H_ 0.93, 1.09 and 1.35), and one methyl group with doublet (δ_H_ 2.03, d, *J* = 1.0 Hz). Further ^1^H–^1^H COSY and HMBC analysis revealed the methyl group with doublet was connected to the trisubstituted double bond (Figure 7), and one hydroxyl group was connected to the methine group C-7 (δ_C_ 76.9, δ_H_ 3.92, d, *J* = 6.5 Hz). The ^1^H–^1^H COSY spectra displayed the following cross-peaks: H-7/H-8 (CH, δ_H_ 2.64, dddd, *J* = 8.5, 8.5, 6.5, 6.5 Hz); H-8/H-9 (CH_2_, δ_H_ 1.45, dd, *J* = 13.5, 8.5 Hz; 1.66, dd, *J* = 13.5, 6.5 Hz), H-8/H-1 (CH, δ_H_ 2.58, dddd, *J* = 10.5, 8.5, 7.5, 2.5 Hz), H-1/H-2 (CH, δ_H_ 2.34, d, *J* = 2.5 Hz), and H-1/H-11 (CH_2_, δ_H_ 1.48, dd, *J* = 12.0, 10.5 Hz; 1.76, dd, *J* = 12.0, 7.5 Hz), so the fragment –CH(–OH) –CH(–CH_2_–)–CH–CH(–CH_2_–)– was established (Figure 7). The HMBC correlations of H-2/C-3, H-2/C-6, H-4/C-5, H-7/C-6, H-9/C-10, H-11/C-10, H-14/C-10, H-15/C-10, H-13/C-5, H-13/C-6, and H-13/C-7, established the planar structure of **5**. The ROESY correlations of H-1/H-8, H-1/H-15, H-2/H-13, H-7/H-8, and H-8/H-15 revealed H-2 and H-13 (CH_3_) have a β-orientation, whereas, H-1, H-7, H-8 and H-15 (CH_3_) have an α-orientation.

**Figure 8:**
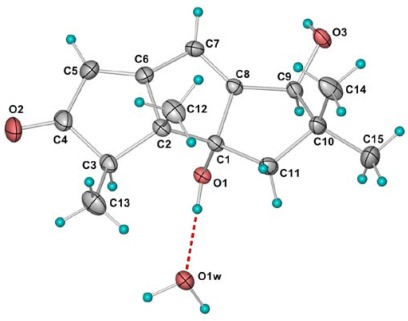
Molecular structure of **6**. Thermal ellipsoids are plotted at 30% probability level.

Compound **6** was identified as hirsutanol C, which was firstly isolated by *Crews *and co-workers from an unidentified fungus [10]. Its NMR data recorded in DMSO-*d*_6_ (Table 1 and Table 2) were slightly different from the reference data recorded in CD_3_OD and dioxane-*d*_8_. The relative configuration was established by single-crystal X-ray diffraction. In the crystal structure of **6**, the molecules related by a simple translation along the *b*-axis are connected by pairs of O3–H…O1 hydrogen bonds to form a ribbon, which is further consolidated by the bridging water molecules with pairs of O1w–H…O3 and O1–H…O1w hydrogen bonds. The composite ribbons are further joined together with O1w–H…O2 hydrogen bonds to form a double layer parallel to the (001) family of planes. Adjacent layers are stacked together with the hydrophobic hydrocarbon skeleton pointing outwards (Figure 8).

**Table 2:** ^1^H NMR data of compounds **1**–**6** (500 MHz, mult., *J *in [Hz]).

Position	1 ^a^	2 ^a^	3 ^a^	4 ^a^	5 ^a^	6 ^b^
1	2.33, dd (10.0, 9.0)				2.58, dddd (10.5, 8.5, 7.5, 2.5)	
2					2.34, d (2.5)	
3		2.99, q (7.0)	2.41, qd (7.0, 1.5)	2.13, qd (7.0, 1.5)		2.77, q (7.0)
4					5.73, q (1.0)	
5	6.02, d (1.5)	6.20, s	α: 2.91, ddd (19.0, 3.5, 1.5); β: 2.32, dd (19.0, 10.0)	α: 2.79, ddd (19.5, 4.5, 1.5); β: 2.62, dd (19.5, 12.0)		5.69, s
6			2.83, ddd (10.0, 7.0, 3.5)	3.12, dd (12.0, 4.5)		
7	α: 2.76, d (15.5); β: 2.71, dd (15.5, 1.5)	7.12, s	4.80, dd (7.0, 1.0)		3.92, d (6.5)	6.32, d (2.5)
8					2.64, dddd (8.5, 8.5, 6.5, 6.5)	
9	α: 1.90, d (14.0); β: 1.65, d (14.0)				α: 1.66, dd (13.5, 6.5); β: 1.45, dd (13.5, 8.5)	4.55 dd (6.0, 2.5)
10						
11	α: 1.76, dd (13.5, 9.0); β: 1.60, dd (13.5, 10.0)	α: 1.97, d(14.0); β: 2.10, d (14.0)	α: 2.38, d (19.0); β: 2.48, dd (19.0, 1.0)	2.78, d (21.0); 2.58, d (21.0)	α: 1.76, dd (12.0, 7.5); β: 1.48, dd (12.0, 10.5)	1.98, d (14.5); 1.52, d (14.5)
12	1.14, s	1.04, s	1.08, s	1.28, s	2.03, d (1.0)	0.89, s
13	5.89, s; 5.16, s	1.16,d (7.0)	1.05, d (7.0)	1.15, d (7.0)	1.35, s	0.93, d (7.0)
14	1.12, s	1.18, s	1.17, s	1.25, s	1.09, s	1.19, s
15	1.19, s	1.40, s	1.16, s	1.20, s	0.93, s	0.80, s
1α-OH		1.94, brs				5.15, s
7β-OH			3.42, brs			
8α-OH	2.05, brs					
9β-OH						5.250, d(6.0)

^a^ Measured in CDCl_3_, and CDCl_3_ was used as an internal standard (δ_H_ 7.26); ^b^ Measured in DMSO-*d*_6_, and DMSO-*d*_6_ was used as an internal standard (δ_H_ 2.50).

Three cancer cell lines: human lung cancer cell line A549, human nasopharyngeal carcinoma cell line CNE2, and human colon cancer cell line LoVo, were used to evaluate the cytotoxic activities of **1**–**6** *in vitro*. As a result, **1** showed potent cytotoxicity against these cancer cell lines with the IC_50_ values of 2.45, 4.95, and 5.47 μM, respectively. In contrast, **2**–**6** were apparently inactive in this assay (IC_50_ > 200 μM).

## 3. Experimental Section

### 3.1. General Experimental Procedures

Preparative HPLC was conducted on a Shimadzu LC-20AT HPLC pump equipped with a SPD-20A dual λ absorbance detector and Shim-pack PRC-ODS HPLC column (250 × 20 mm). Melting points were measured on an X-6 micro-melting-point apparatus (Beijing Fukai Science and Technology Development, Beijing, China) and were uncorrected. Optical rotations were acquired using a Schmidt and Haensch polartronic HNQW5 optical rotation spectrometer. IR spectra were recorded on a Nicolet Avatar 330 FT-IR spectrophotometer. UV spectra were recorded on a Shimadzu UV-Vis-NIR spectrophotometer. 1D and 2D NMR spectra were recorded on a Varian Inova-500 spectrometer and Bruker Avancell-400 spectrometer. The chemical shifts were referenced to the residual solvent signal (CDCl_3_: δ_H_ 7.26 and δ_C_ 77.0; DMSO-*d*_6_: δ_H_ 2.50 and δ_C_ 39.43). HPLC-MS analyses were performed with Thermo Finnigan LCQ^TM^ DECA XP liquid chromatography-mass spectrometry. Mass spectra were obtained on Thermo DSQ EI low resolution mass spectrometer and Thermo MAT95XP EI high resolution mass spectrometer. X-ray diffraction data were acquired on a Bruker SMART 1000 CCD X-ray single crystal diffractometer.

### 3.2. Fungal Strain and Culture Method

*Chondrostereum *sp. was isolated from the inner tissue of soft coral *Sarcophyton tortuosum* collected from Hainan Sanya National Coral Reef Reserve, China. This fungal strain was maintained on potato dextrose agar (PDA) slants. Fermentation medium was potato dextrose broth (PDB, potatoes 200 g, dextrose 20 g, seawater 1 L). Plugs of agar supporting mycelia growth were cut and transferred aseptically to a 500 mL Erlenmeyer flask containing 200 mL PDB liquid medium. The liquid medium was sterilized at 120 °C for 30 min. The flask was incubated at 28 °C on a rotary shaker (120 rpm) for 5 days. The mycelia were aseptically transferred to 500 mL Erlenmeyer flasks containing 200 mL of the same liquid medium. The flasks were incubated at 28 °C on a rotary shaker (120 rpm) for 20 days. 

### 3.3. Extraction and Isolation

60 L of grown culture broth was filtered through cheesecloth. The culture broth was successively extracted three times with EtOAc. The EtOAc extract was concentrated by low-temperature rotary evaporation. The extract (24.6 g) was chromatographed on a silica gel column with petroleum ether-EtOAc (100:0–0:100) and then EtOAc-MeOH (100:0–0:100) as the eluent to afford 18 fractions (code Fr. 1–Fr. 18). Fr. 9 was further purified by RP-HPLC with a gradient of H_2_O-MeCN (40:60 up to 0:100, v/v) to afford compound **1 **(48 mg). Fr. 10–12 was further purified by Sephadex LH-20 gel column chromatography and repeated RP-HPLC, eluted with H_2_O-MeCN (60:40, v/v) to yield compounds **2 **(39 mg) and **5** (21 mg). Compounds **3** (59 mg) and **4** (32 mg) were obtained from Fr. 13 by repeated RP-HPLC using H_2_O-MeCN (70:30, v/v) as eluent. Fr. 14–15 was purified by repeated silica gel column chromatography, followed by recrystallization from EtOAc solution, to afford compound **6** (46 mg).

Chondrosterin A (**1**): Yellowish oil; [α]^20^_D_ +112 (*c* 0.024, MeOH); UV (MeOH) λ_max_ (log ε) 249 nm (4.06); IR (KBr) *v*_max_ 3433, 2951, 2867, 1693, 1622, 1462, 1377, 1312, 1259, 1211, 1156, 1123, 1037, 941, 864 cm^−1^; ^1^H and ^13^C NMR data, see Table 1; EIMS *m/z* 232, 217, 176, 122, 91, 92, 77, 55; HREIMS *m/z *232.1456 [M]^+^ (calcd for C_15_H_20_O_2_, 232.1458).

Chondrosterin B (**2**): Yellowish oil; [α]^20^_D_ +154 (*c* 0.022, MeOH); UV (MeOH) λ_max_ (log ε) 301 nm (4.07); IR (KBr) *v*_max_ 3423, 2969, 2872, 1698, 1616, 1590, 1463, 1379, 1312, 1256, 1191, 1142, 1115, 1069, 998, 903 cm^–1^; ^1^H and ^13^C NMR data in CDCl_3_, see Table 1; ^1^H NMR (400 MHz, DMSO-*d*_6_) δ: 7.25 (s, H-7), 6.20 (s, H-5), 5.52 (s, 1α-OH), 2.87 (q, *J* = 7.0 Hz, H-3), 2.01 (d, *J* = 14.0 Hz, H-11β), 1.89 (d, *J* = 14.0 Hz, H-11α), 1.30 (s, H-15), 1.09 (s, H-14), 1.03 (d, *J* = 7.0 Hz, H-13), 0.94 (s, H-12). EIMS *m/z* 246, 231, 218, 203, 190, 175, 162, 147, 134, 119, 105, 91, 77; HREIMS *m/z* 246.1250 [M]^+^ (calcd for C_15_H_18_O_3_, 246.1250).

Chondrosterin C (**3**): White solid; mp 130–131 °C; [α]^20^_D_ −177 (*c* 0.034, MeOH); UV (MeOH) λ_max_ (log *ε*) 236 nm (3.83); IR (KBr) *v*_max_ 3355, 2968, 2958, 2938, 2910, 1734, 1684, 1635, 1460, 1429, 1388, 1370, 1315, 1288, 1224, 1174, 1096, 1075, 1047, 995 cm^−1^; ^1^H and ^13^C NMR data, see Table 1; EIMS *m/z* 248, 233, 230, 215, 205, 189, 177, 173, 163, 159, 145, 135, 119, 105, 91, 77, 55; HREIMS *m/z *248.1405 [M]^+^(calcd for C_15_H_20_O_3_, 248.1407). 

Chondrosterin D (**4**): Colorless crystals (ethyl acetate); mp 263–264 °C; [α]^20^_D_ −300 (*c* = 0.025, MeOH); UV (MeOH) λ_max_ (log ε) 305 nm (2.34), 261 nm (3.36), 222 nm (3.38); IR (KBr) *v*_max_ 2975, 2927, 1737, 1687, 1610, 1454, 1420, 1391, 1336, 1322, 1047, 1027 cm^–1^; ^1^H and ^13^C NMR data, see Table 1; EIMS *m/z *246, 231, 218, 203, 189, 175, 161, 147, 135, 119, 105, 91, 83, 77, 65, 55; HREIMS *m/z *246.1255 [M]^+^ (calcd for C_15_H_18_O_3_, 246.1250).

Chondrosterin E (**5**): White solid; mp 84–85 °C; [*α*]^20^_D_ +66 (*c* 0.028, MeOH); UV (MeOH) λ_max_ (log ε) 235 nm (3.81); IR (KBr) *v*_max_ 3463, 2950, 2927, 2865, 1678, 1613, 1440, 1378, 1361, 1314, 1259, 1170, 1096, 1065, 1011, 954, 876, 852 cm^−1^; ^1^H and ^13^C NMR data, see Table 1; EIMS *m/z* 234, 216, 206, 201, 173, 135, 110, 95, 91, 79, 69, 55; HREIMS *m/z *234.1613 [M]^+^ (calcd for C_15_H_22_O_2_, 234.1614).

### 3.4. Crystal Structure Determination of 4 and 6

Crystals of **4** and **6** were obtained from EtOAc solution. Chondrosterin D (**4**): C_15_H_18_O_3_, *M* = 246.29, colorless block, orthorhombic system, space group *P*2_1_2_1_2_1_, *a *= 6.5259(11), *b* = 12.735(2), *c* = 16.396(3) Å, *V* = 1362.6(4) Å^3^, *Z* = 4, *d* = 1.201 g/cm^3^, crystal size 0.40 × 0.38 × 0.36 mm^3^. Hirsutanol C (**6**): C_15_H_20_O_3_·H_2_O, *M* = 266.34, colorless block, monoclinic system, space group *P*2_1_, *a *= 8.0825(19), *b* = 8.912(2), *c* = 10.695(3) Å, *V* = 734.1(3) Å^3^, *Z* = 2, *d* = 1.205 g/cm^3^, crystal size 0.42 × 0.40 × 0.38 mm^3^. X-ray diffraction data were collected on a Bruker SMART 1000 CCD diffractometer with Mo *K*_α_ radiation (λ = 0.71073Å) at room temperature. The data were processed using CrysAlis. The structures were solved by direct method. H-atoms were added in ideal positions and refined as riding models. The structures were refined using full-matrix least-squares based on *F*^2^ with program SHELXL [11,12]. 

CCDC 847843 and 847844 contain the supplementary crystallographic data of compounds **4** and **6** respectively [13].

### 3.5. Cytotoxicity Assay

The *in vitro* cytotoxicity of **1**–**6** was determined by means of the colorimetric MTT (3-(4,5-dimethylthiazol-2-yl)-2,5-diphenyl-2*H*-tetrazolium bromide) assay. The tested human cancer cell lines were seeded in 96-well plates at a density of 3 × 10^7^ cells/L, and the compounds were added at various concentrations (0.125–50 mg/L). After 48 h, MTT was added to the culture medium at a final concentration of 0.5 mg/mL, and the plates were incubated for 4 h at 37 °C. The supernatant was removed. The formazan crystals were dissolved in DMSO (150 μL) with gentle shaking at room temperature. The absorbance at 570 nm was recorded with a microplate reader (Bio-Rad, USA), and the data were analyzed with the SPSS [14]. 

## 4. Conclusions

The marine fungus *Chondrostereum* sp. was cultured in PDB medium and afforded five new sesquiterpenoids, chondrosterins A–E (**1**–**5**), and the known compound hirsutanol C (**6**). **1**–**4 **and **6** are hirsutane-type sesquiterpenoids, **5** has a novel rearranged hirsutane skeleton, which could be derived by migration of a methyl group from C-2 to C-6. Chondrosterin A (**1**), with the typical α-methylene ketone group, showed significant cytotoxic activities. These results indicated the metabolites produced by *Chondrostereum* sp. in PDB medium were different from those in GPY medium. By altering the fermentation conditions, e.g. carbon and nitrogen source, inorganic salts, *Chondrostereum* sp. can produce highly functionalized hirsutane derivatives with a surprising chemodiversity. Furthermore, the metabolites isolation work based on ^13^C NMR screening seems effectively to obtain the novel hirsutane-type compounds containing carbonyl groups.

## Supplementary Files

Supplementary File 1: PDF-Document (PDF, 1401 KB)
