# Supplementary material for: Chondrosterins A–E, Triquinane-Type Sesquiterpenoids from Soft Coral-Associated Fungus Chondrostereum sp
Source: Mar Drugs. 2012 Mar 13;10(3):627–38. doi: 10.3390/md10030627 (PMC3347020; doi:10.3390/md10030627)
Supplement: Supplementary File 1: — PDF-Document (PDF, 1401 KB) [file marinedrugs-10-00627-s001.pdf]

Supporting Information

Chondrosterins A–E，Triquinane-Type Sesquiterpenoids from Soft Coral-Associated Fungus *Chondrostereum* sp.

Hou-Jin Li [1], Ying-Lu Xie [1], Zhong-Liang Xie [2], Ying Chen [1], Chi-Keung Lam [1] and Wen-Jian Lan[2,*]

[1] School of Chemistry and Chemical Engineering, Sun Yat-sen University, Guangzhou 510275, P. R. China; E-Mails: ceslhj@mail.sysu.edu.cn(H.L.); 359160016@qq.com(Y.X.); 540170598@qq.com(Y.C.); cklam@mail.sysu.edu.cn(C. L.)

[2] School of Pharmaceutical Sciences, Sun Yat-sen University, Guangzhou 510006, P. R. China; E-Mails: 908129138@qq.com (Z.X.); lanwj@mail.sysu.edu.cn (W.L.)

***** Author to whom correspondence should be addressed; E-Mail: lanwj@mail.sysu.edu.cn (W. L.); Tel.: +86-20-39943042; Fax: +86-20-39943000.

Table of Contents

S1: HREIMS of chondrosterin A (**1**)

S2: ^{1}H NMR (500 MHz, $CDCl_3$) spectrum of chondrosterin A (**1**)

S3: ^{13}C NMR (125 MHz, $CDCl_3$) spectrum of chondrosterin A (**1**)

S4: gHMQC of chondrosterin A (**1**)

S5: gHMBC of chondrosterin A (**1**)

S6: ^{1}H–^{1}H gCOSY of chondrosterin A (**1**)

S7: ROESY of chondrosterin A (**1**)

S8: HREIMS of chondrosterin B (**2**)

S9: ^{1}H NMR (500 MHz, $CDCl_3$) spectrum of chondrosterin B (**2**)
S10: ^{13}C NMR (125 MHz, $CDCl_3$) spectrum of chondrosterin B (**2**)
S11: gHMQC of chondrosterin B (**2**)
S12: gHMBC of chondrosterin B (**2**)
S13: ^{1}H NMR (400 MHz, [D6]DMSO) spectrum of chondrosterin B (**2**)
S14: ROESY of chondrosterin B (**2**) (400 MHz, [D6]DMSO)
S15: HREIMS of chondrosterin C (**3**)
S16: ^{1}H NMR (500 MHz, $CDCl_3$) spectrum of chondrosterin C (**3**)
S17: ^{13}C NMR (125 MHz, $CDCl_3$) spectrum of chondrosterin C (**3**)
S18: gHMQC of chondrosterin C (**3**)
S19: gHMBC of chondrosterin C (**3**)
S20: ^{1}H–^{1}H gCOSY of chondrosterin C (**3**)
S21: NOE of chondrosterin C (**3**)
S22: HREIMS of chondrosterin D (**4**)
S23: ^{1}H NMR (500 MHz, $CDCl_3$) spectrum of chondrosterin D (**4**)
S24: ^{13}C NMR (125 MHz, $CDCl_3$) spectrum of chondrosterin D (**4**)
S25: gHMQC of chondrosterin D (**4**)
S26: gHMBC of chondrosterin D (**4**)
S27: HREIMS of chondrosterin E (**5**)
S28: ^{1}H NMR (500 MHz, $CDCl_3$) spectrum of chondrosterin E (**5**)
S29: ^{13}C NMR (125 MHz, $CDCl_3$) spectrum of chondrosterin E (**5**)
S30: gHMQC of chondrosterin E (**5**)
S31: gHMBC of chondrosterin E (**5**)
S32: ^{1}H–^{1}H gCOSY of chondrosterin E (**5**)
S33: ROESY of chondrosterin E (**5**)

Full ms [227.500 - 246.500] - Range: 232.000 - 232.500

Scan No. 11 of 15

Scan #: 11

RT: 0.44

Data points: 1

Mass	Relative Intensity	Theoretical Mass	Delta [ppm]	Delta [mmu]	RDB	Composition
232.1456	100.0	232.1458	-0.7	-0.2	6.0	C_{15} H_{20} O_2

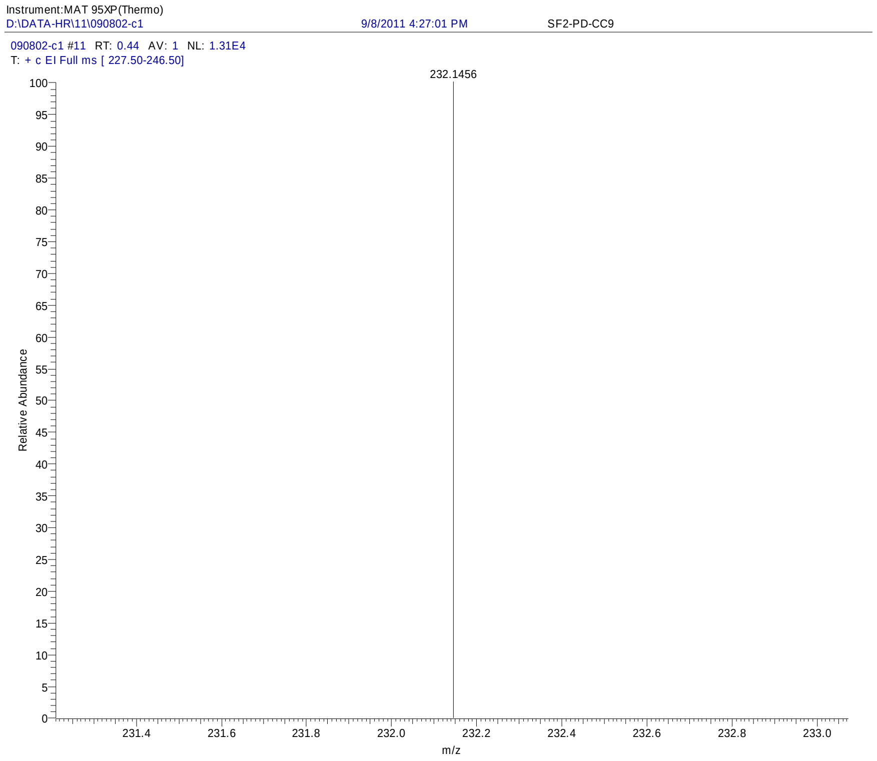

S1: HREIMS of chondrosterin A (**1**)

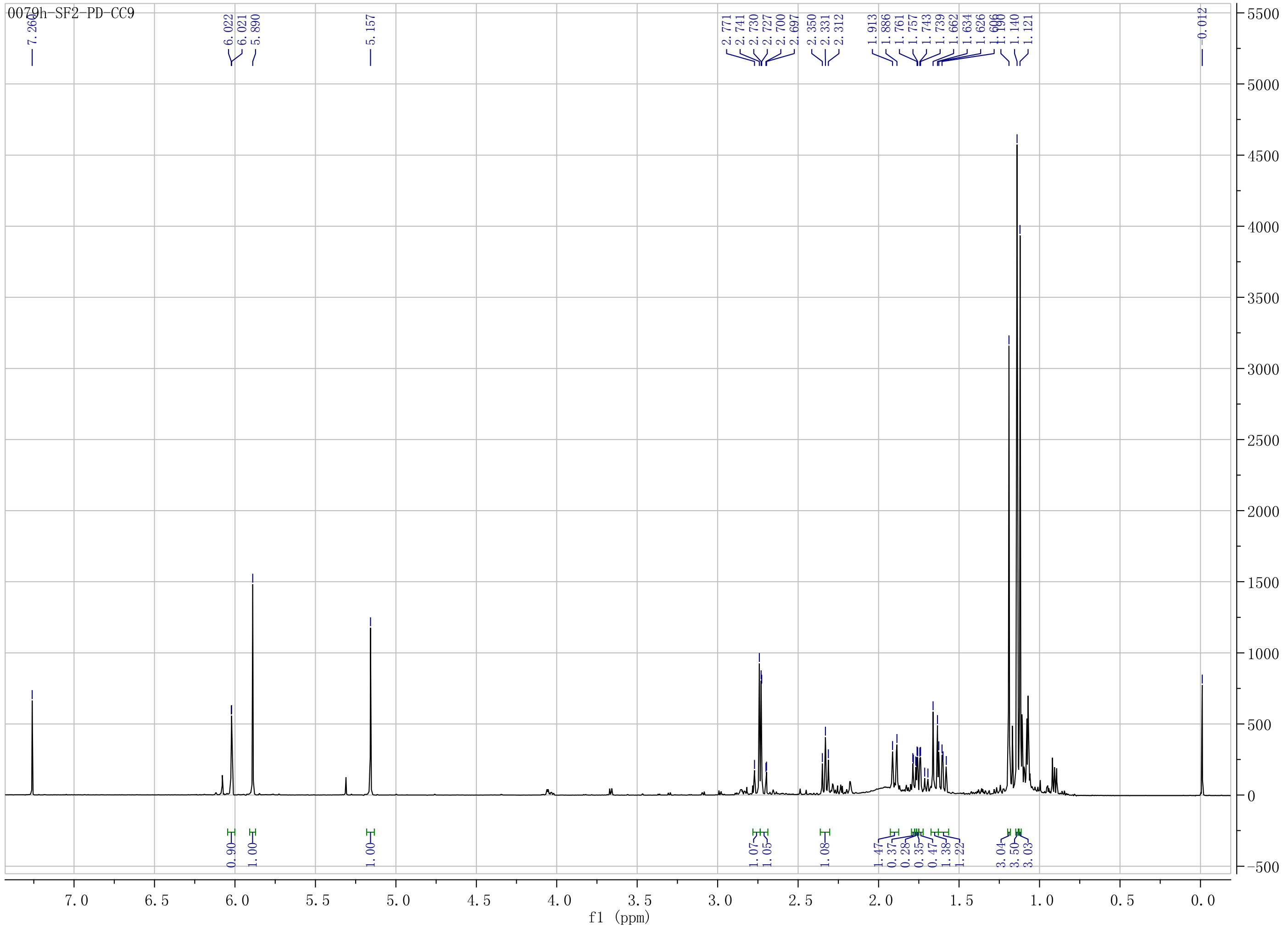

S2: ^{1}H NMR (500 MHz, $CDCl_3$) spectrum of chondrosterin A (**1**)

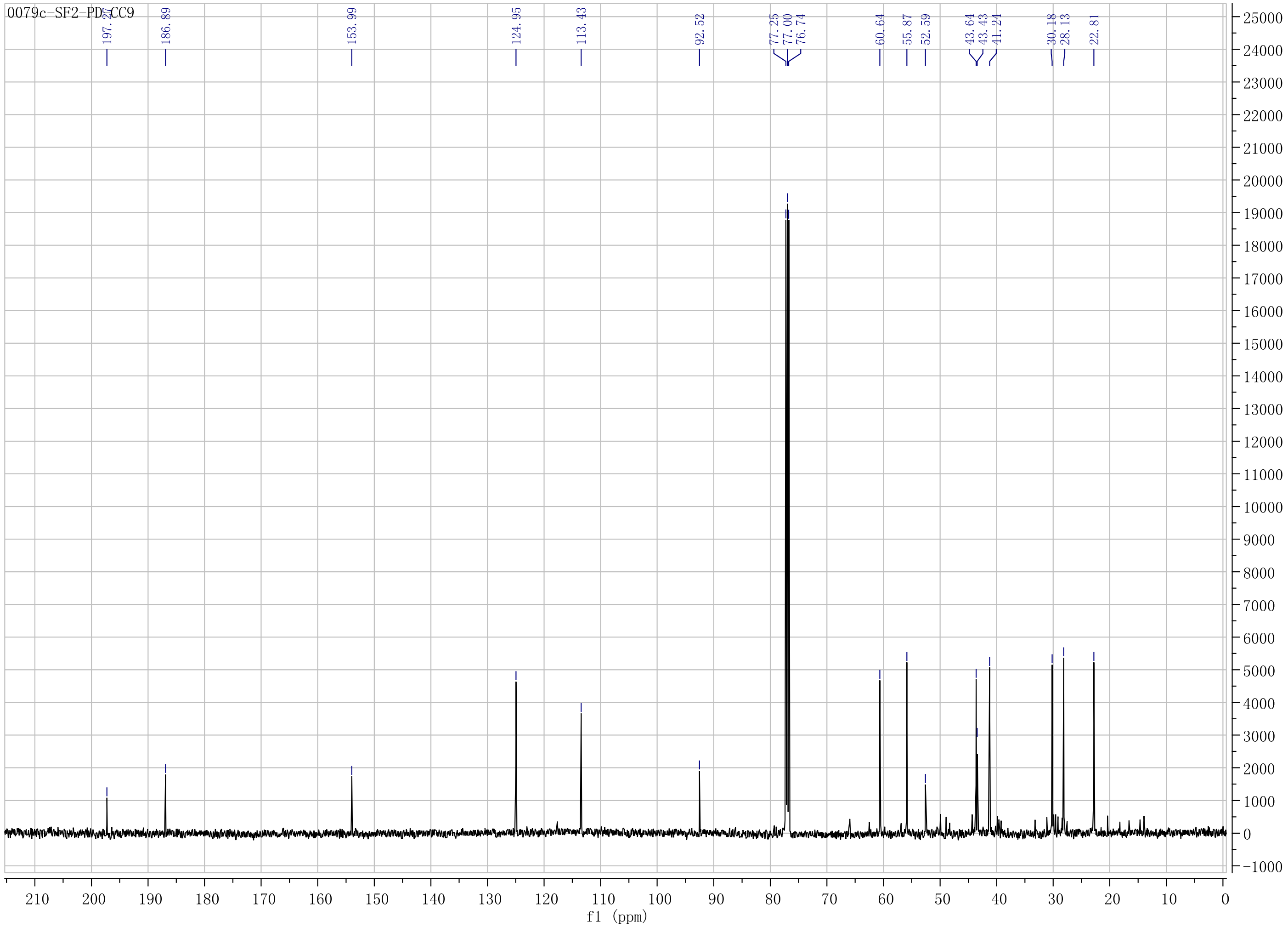

S3: ^{13}C NMR (125 MHz, $CDCl_3$) spectrum of chondrosterin A (**1**)

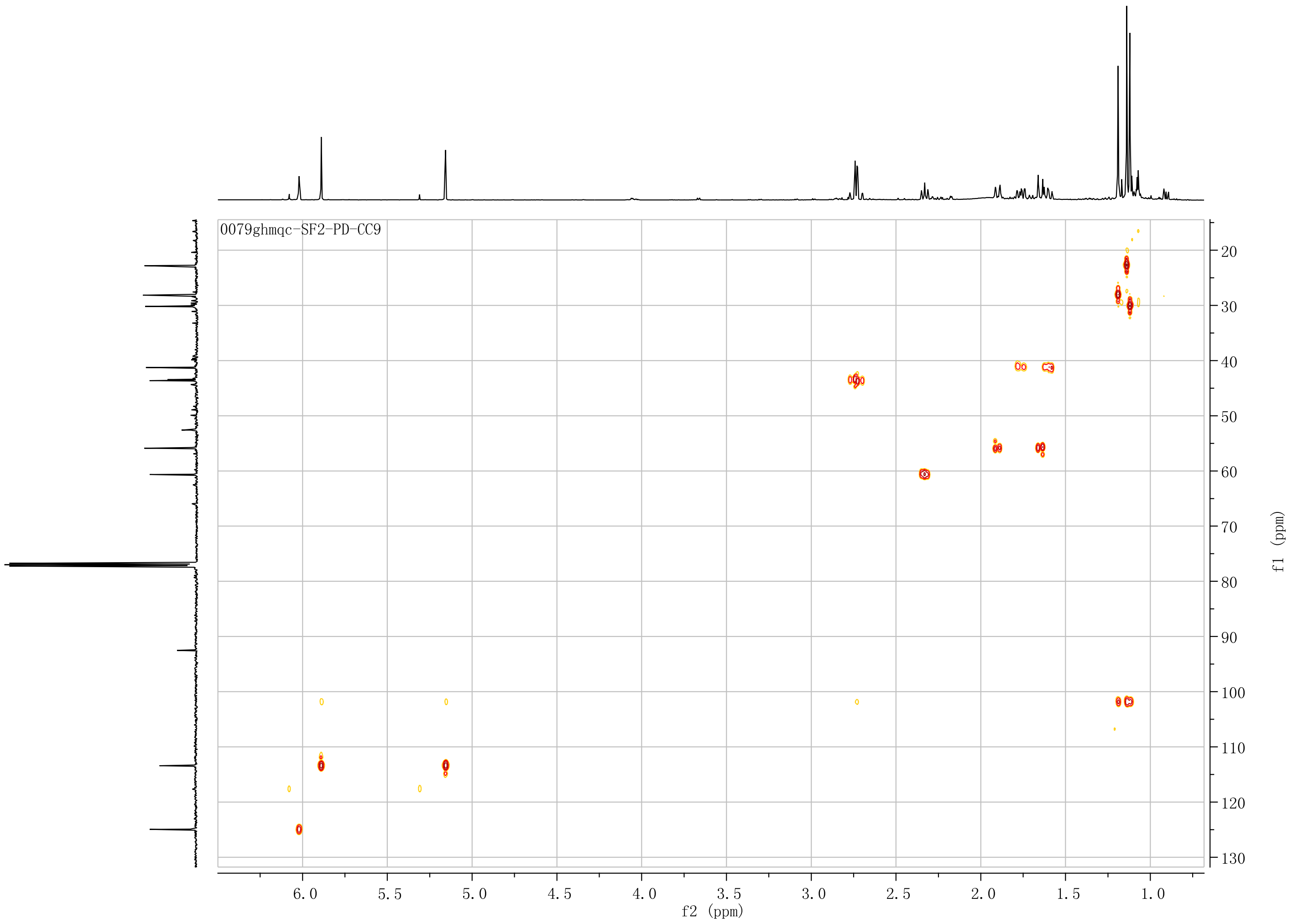

S4: gHMQC of chondrosterin A (**1**)

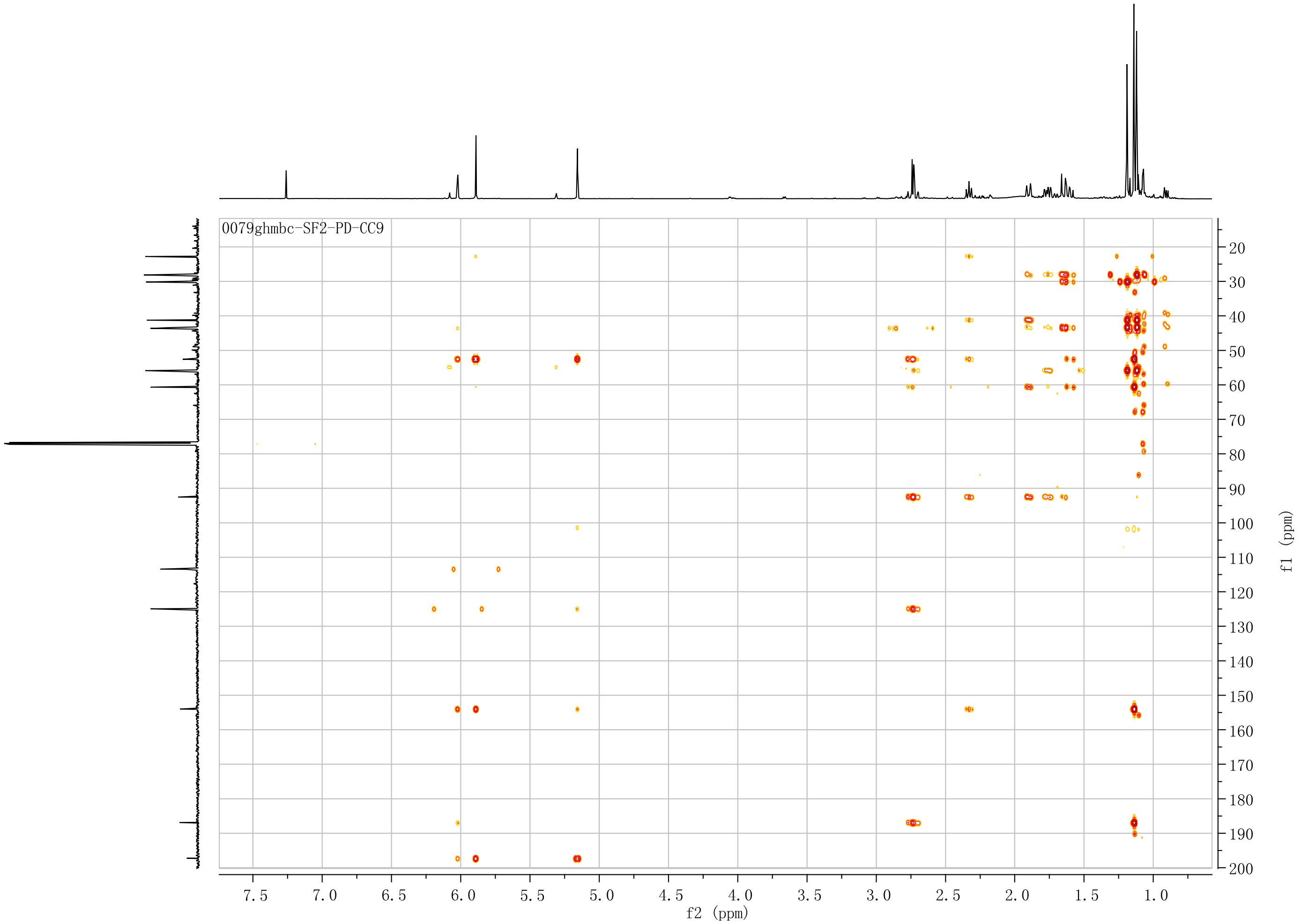

S5: gHMBC of chondrosterin A (**1**)

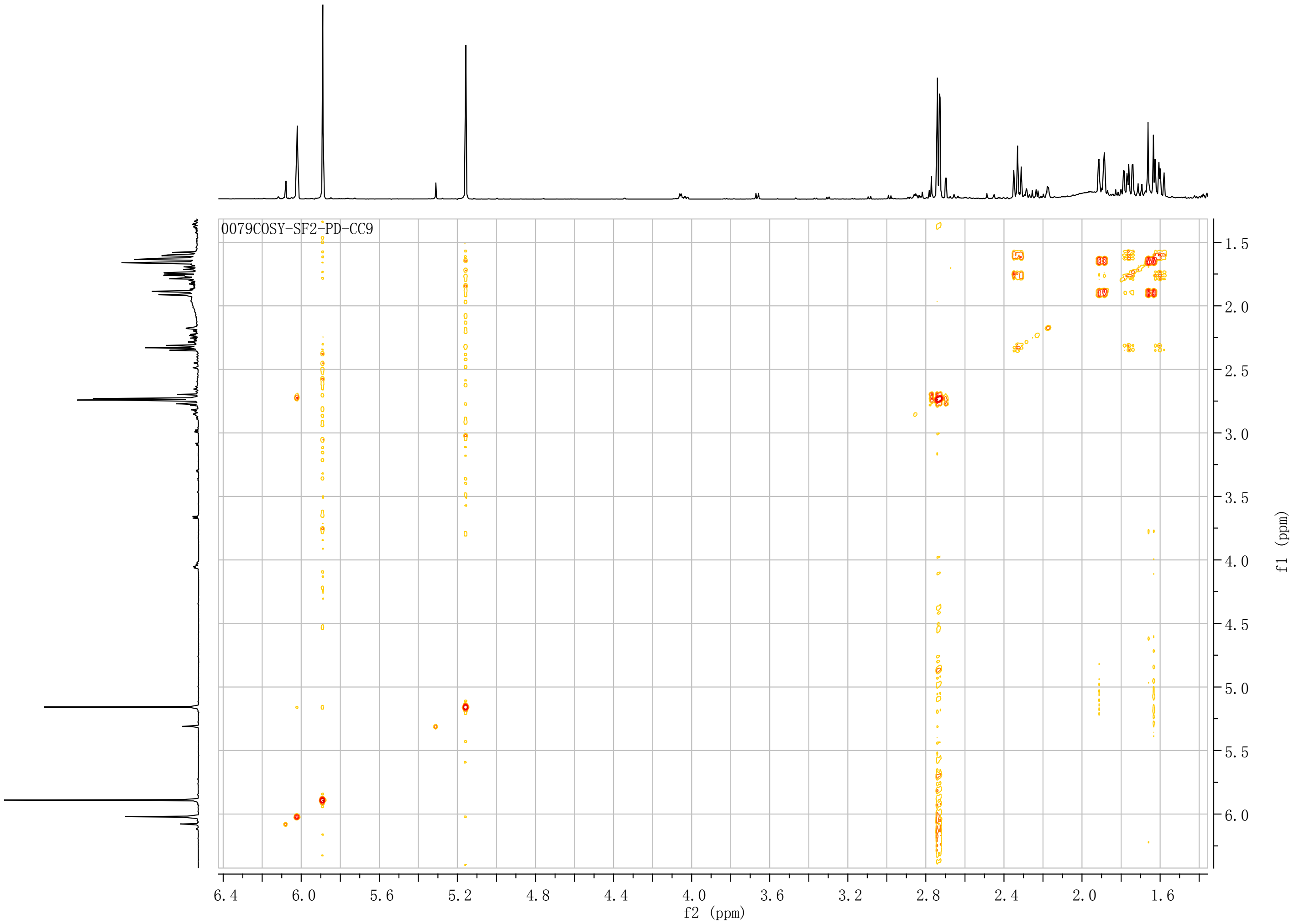

S6: ^{1}H–^{1}H gCOSY of chondrosterin A (**1**)

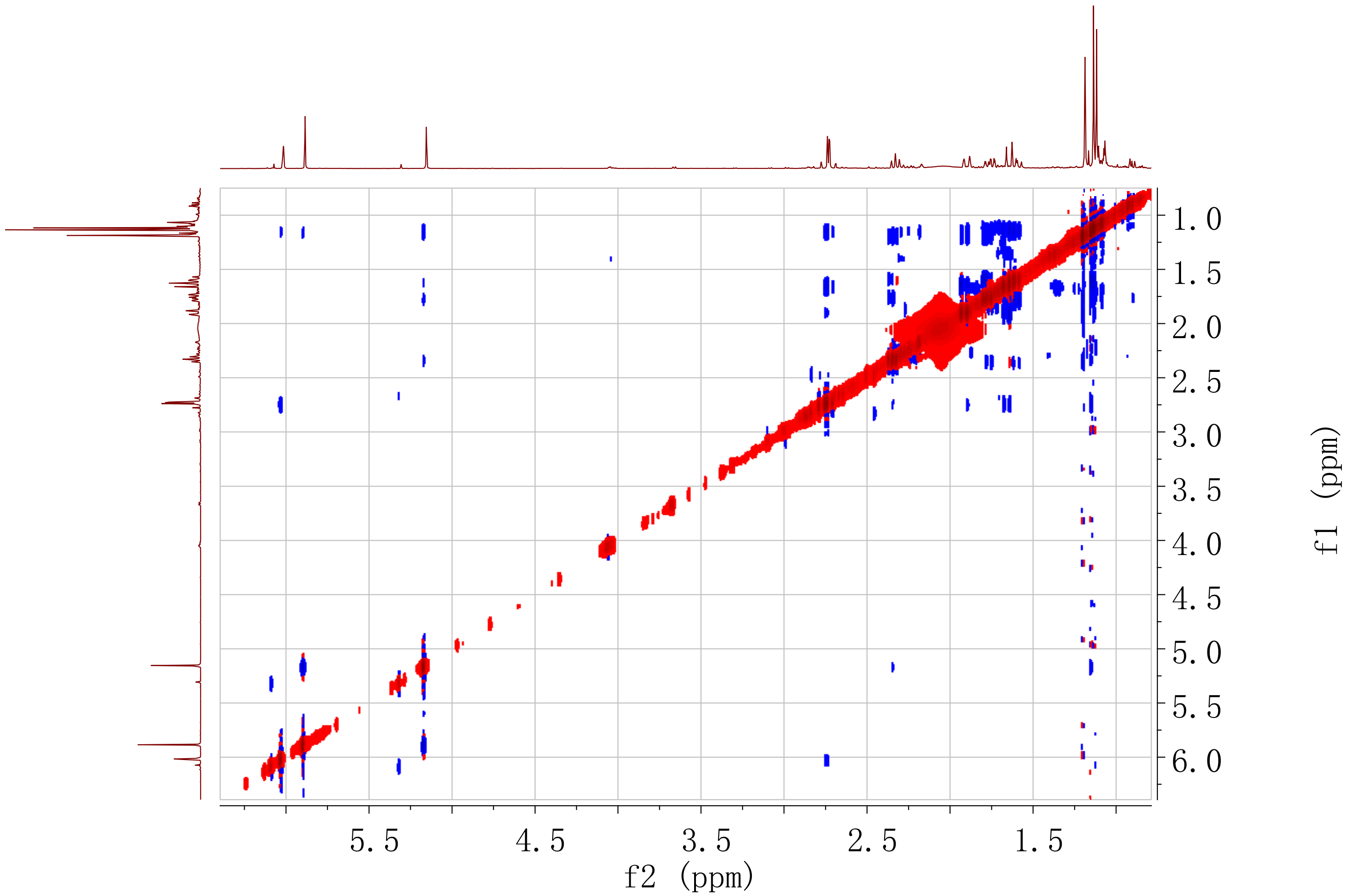

S7: ROESY of chondrosterin A (**1**)

Full ms [239.500 - 258.500] - Range: 246.000 - 246.500

Scan No. 16 of 16

Scan #: 16

RT: 0.60

Data points: 1

Mass	Relative Intensity	Theoretical Mass	Delta [ppm]	Delta [mmu]	RDB	Composition
246.1250	77.1	246.1250	-0.3	-0.1	7.0	C_{15} H_{18} O_3

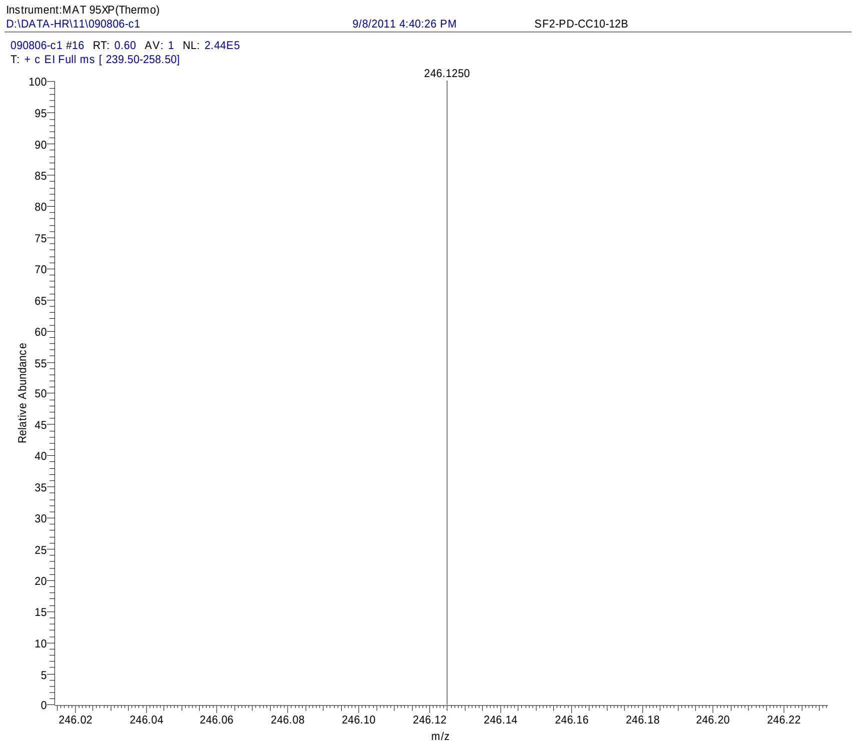

S8: HREIMS of chondrosterin B (**2**)

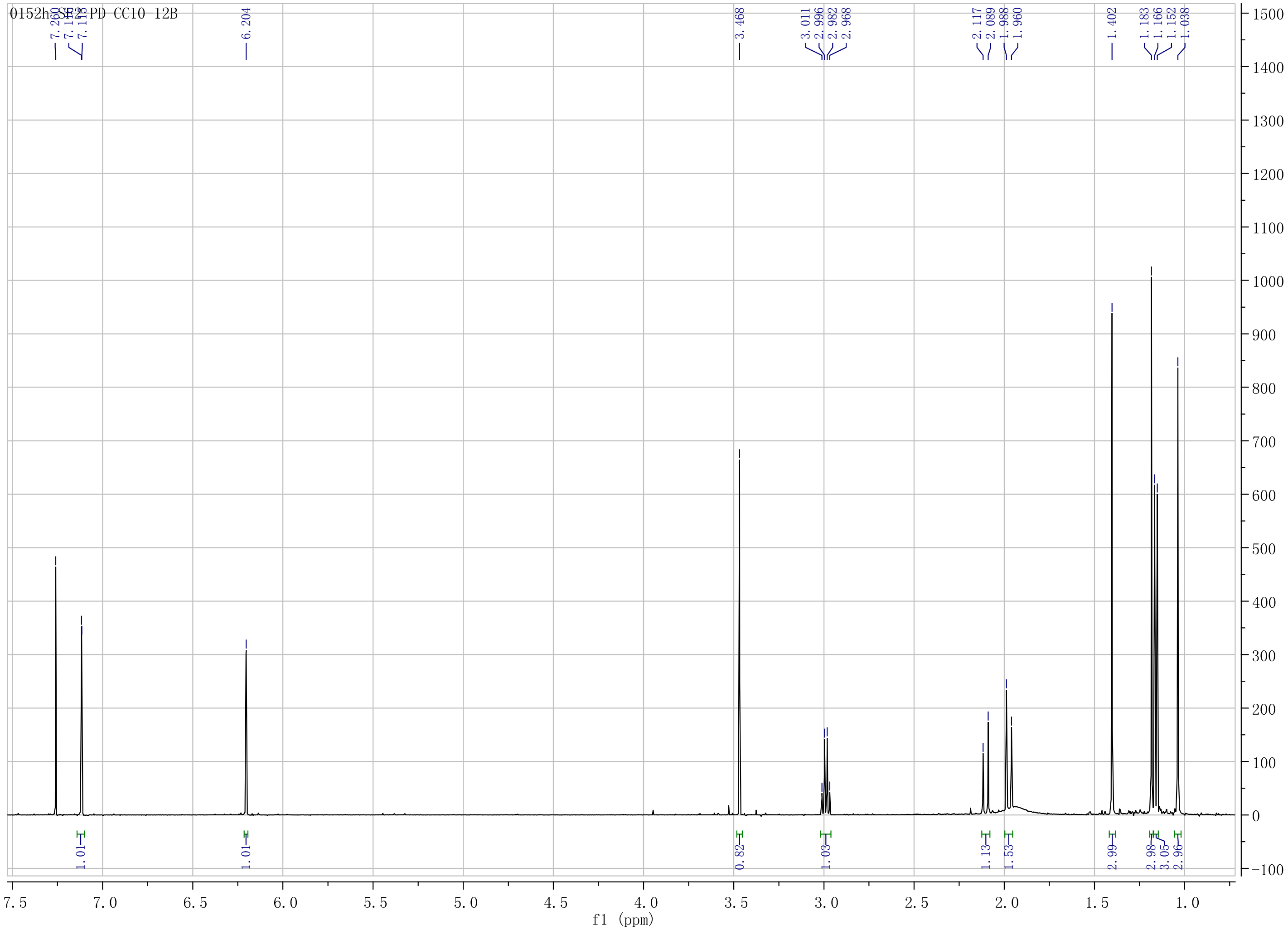

S9: ^{1}H NMR (500 MHz, $CDCl_3$) spectrum of chondrosterin B (**2**)

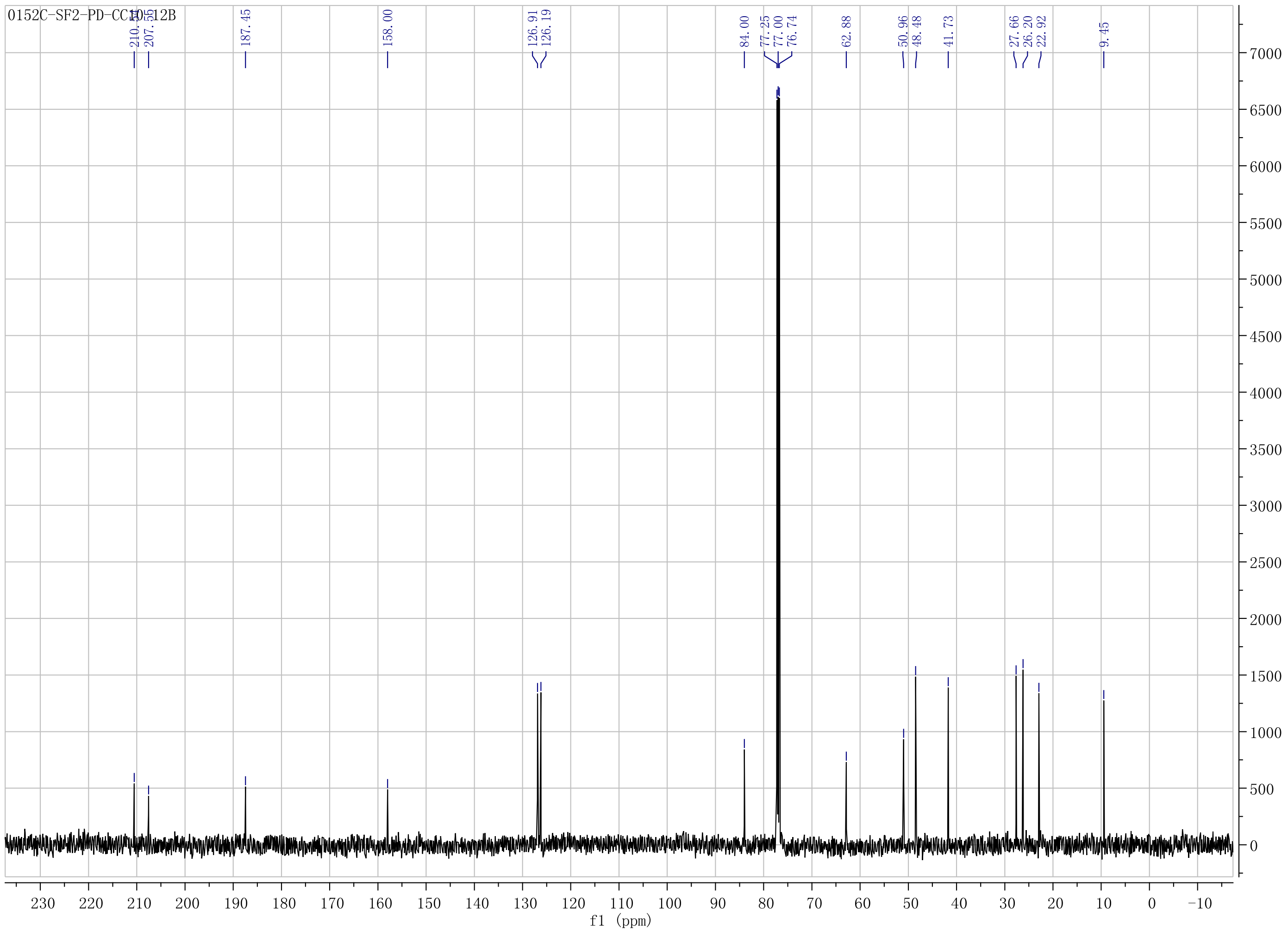

S10: ^{13}C NMR (125 MHz, $CDCl_3$) spectrum of chondrosterin B (**2**)

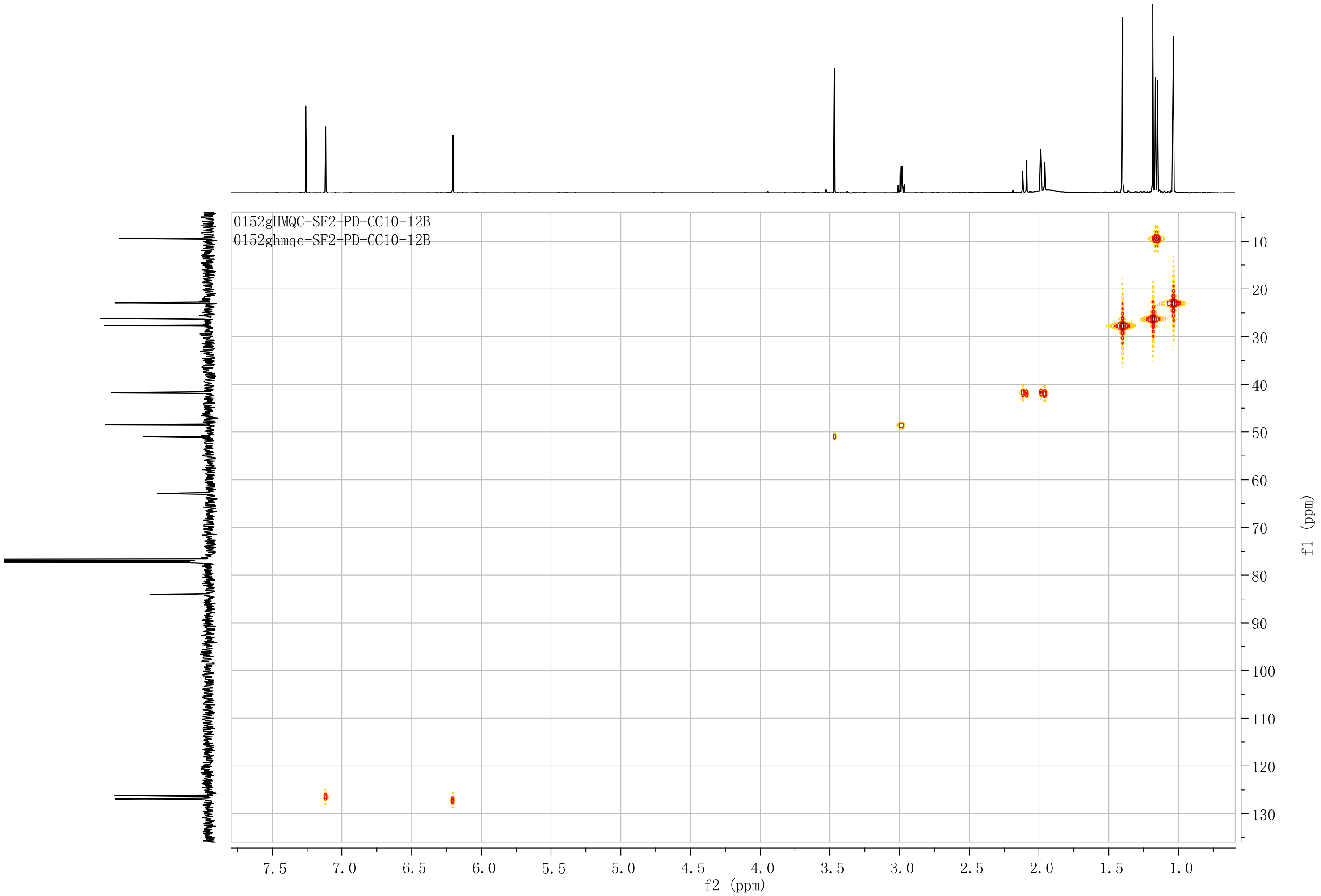

S11: gHMQC of chondrosterin B (**2**)

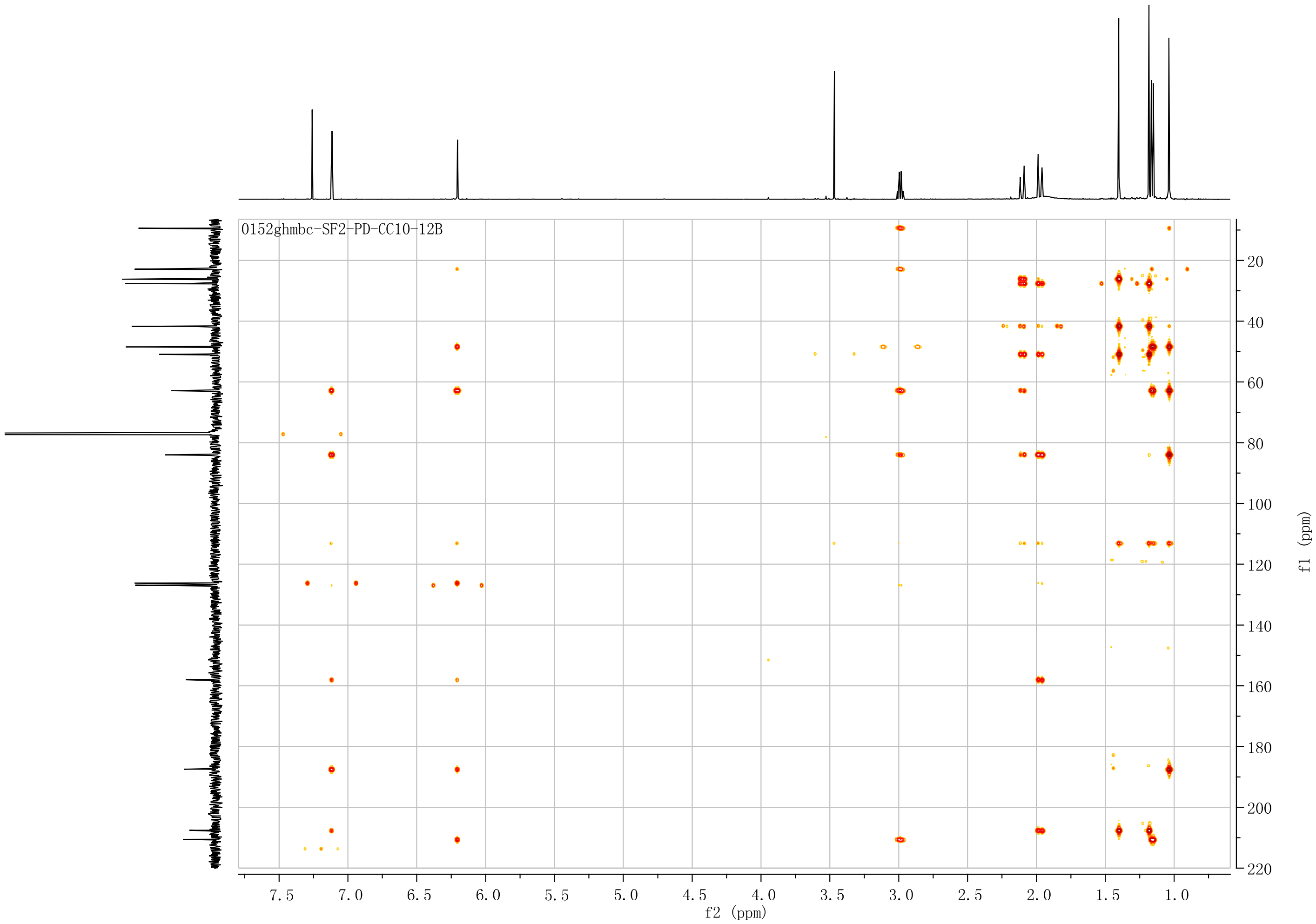

S12: gHMBC of chondrosterin B (**2**)

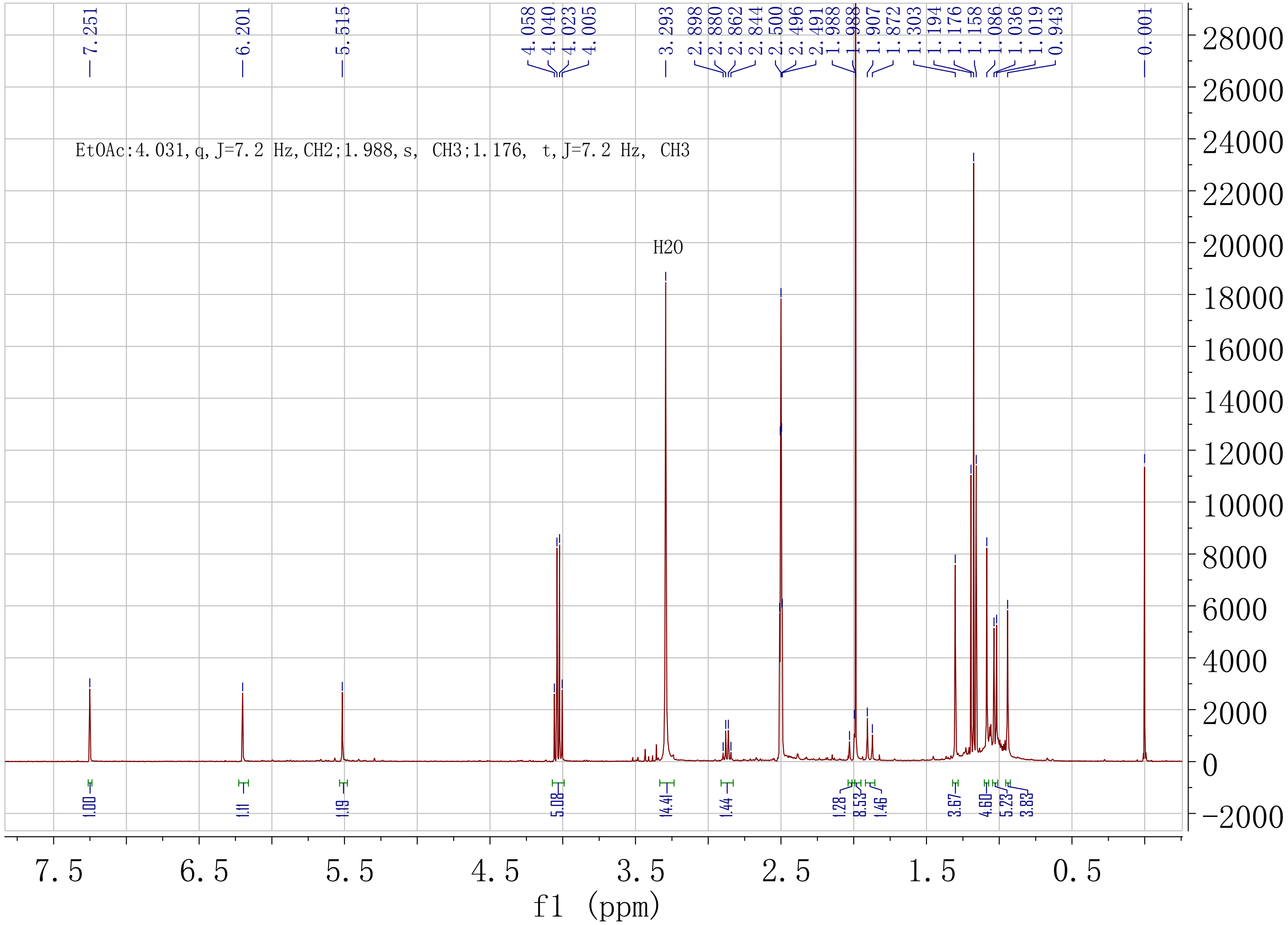

S13: ^{1}H NMR (400 MHz, [D6]DMSO) spectrum of chondrosterin B (**2**)

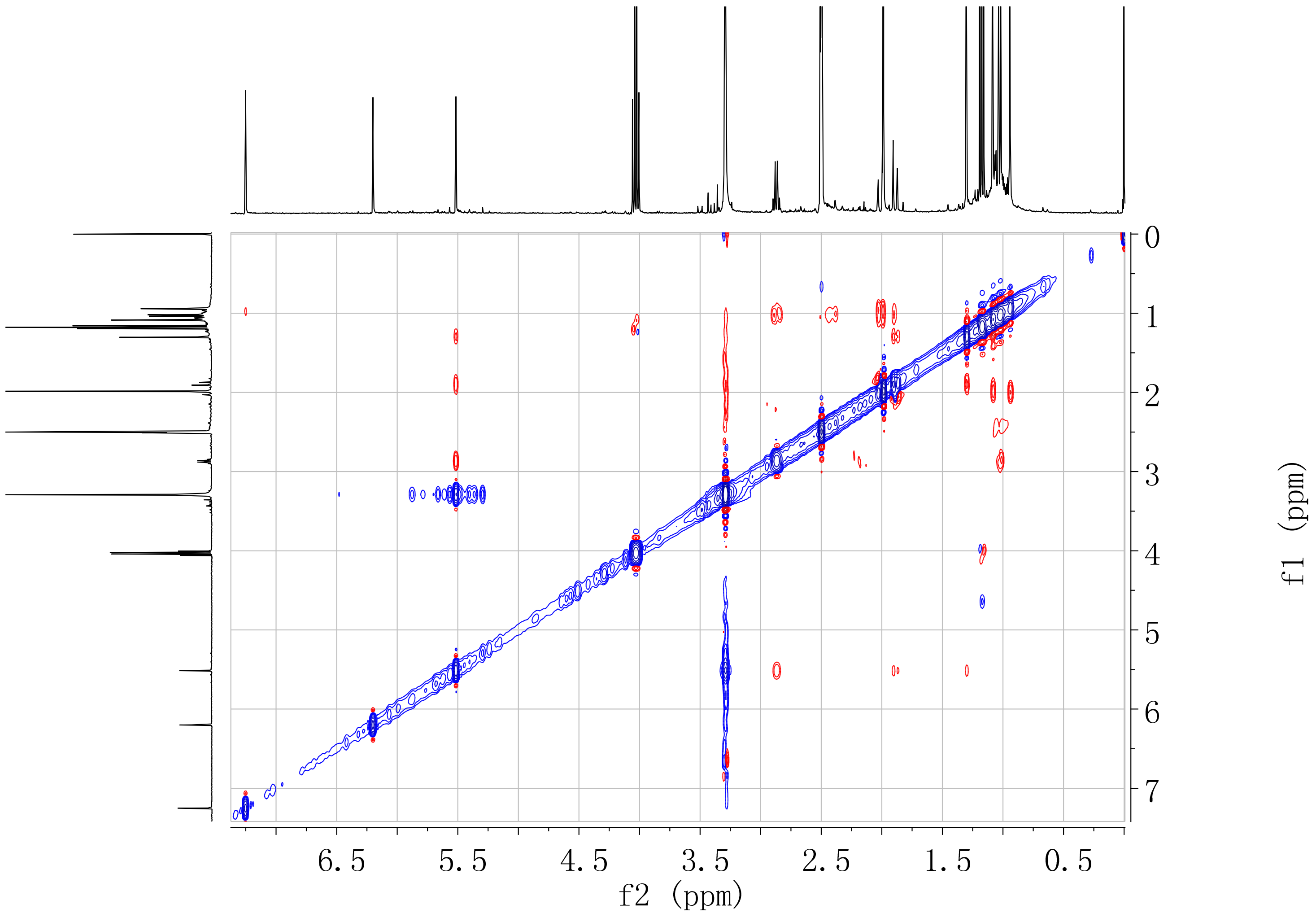

S14: ROESY of chondrosterin B (**2**) (400 MHz, [D6]DMSO)

Full ms [239.500 - 258.500] - Range: 248.000 - 248.300

Scan No. 13 of 18

Scan #: 13

RT: 0.51

Data points: 1

Mass	Relative Intensity	Theoretical Mass	Delta [ppm]	Delta [mmu]	RDB	Composition
248.1405	100.0	248.1407	-0.9	-0.2	6.0	C_{15} H_{20} O_{3}

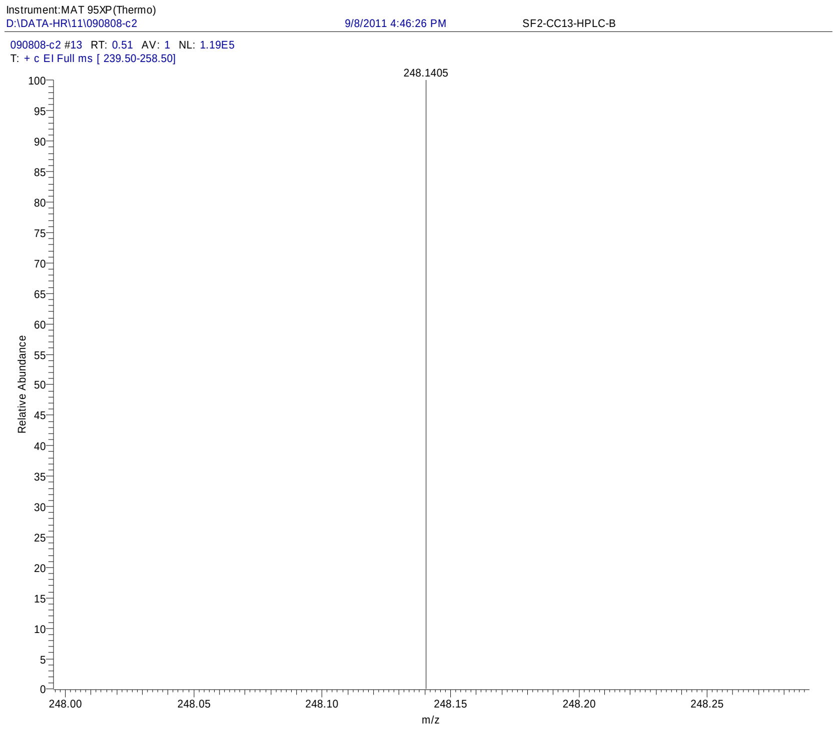

S15: HREIMS of chondrosterin C **(3)**

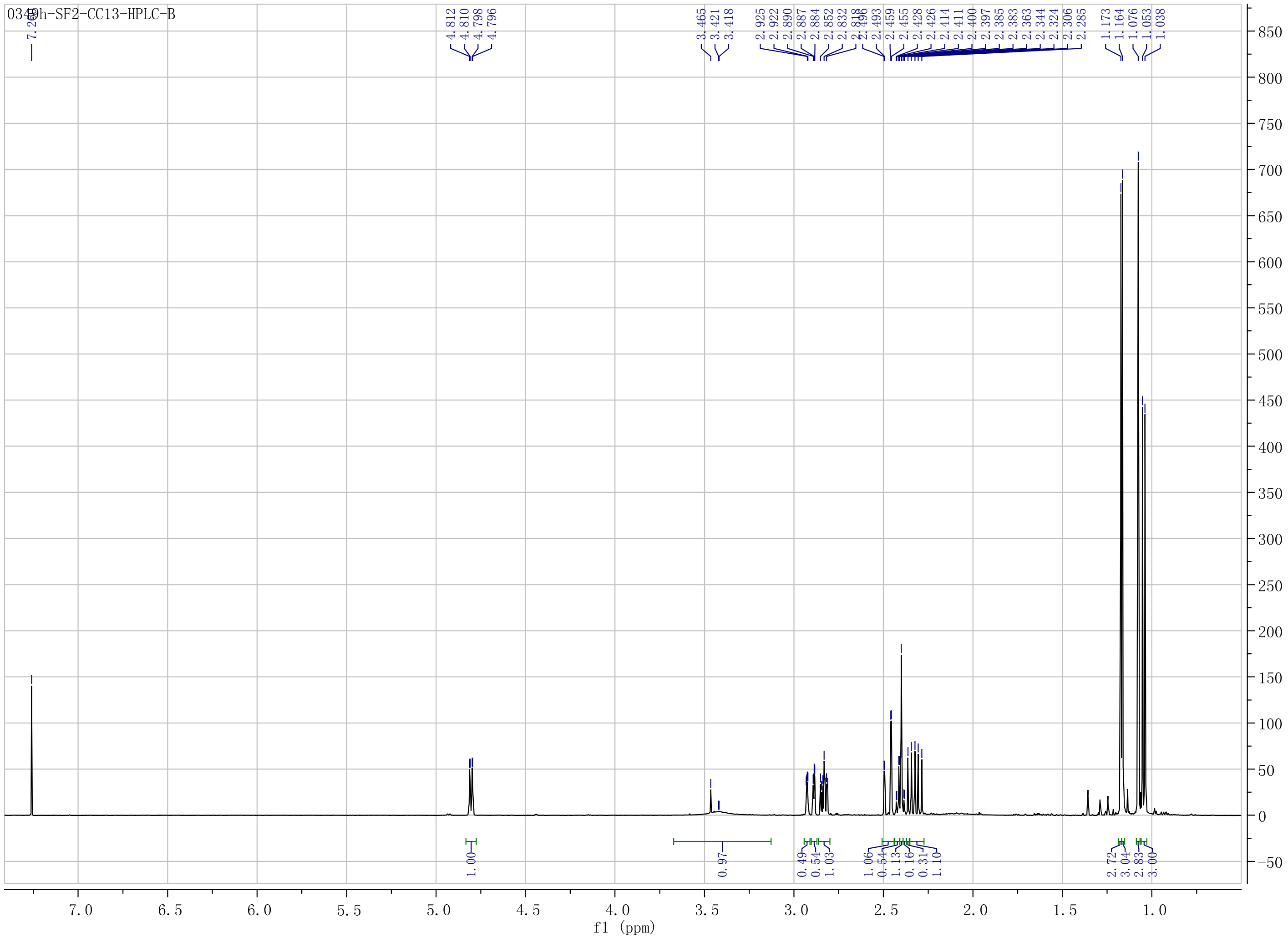

S16: ^{1}H NMR (500 MHz, $CDCl_3$) spectrum of chondrosterin C (**3**)

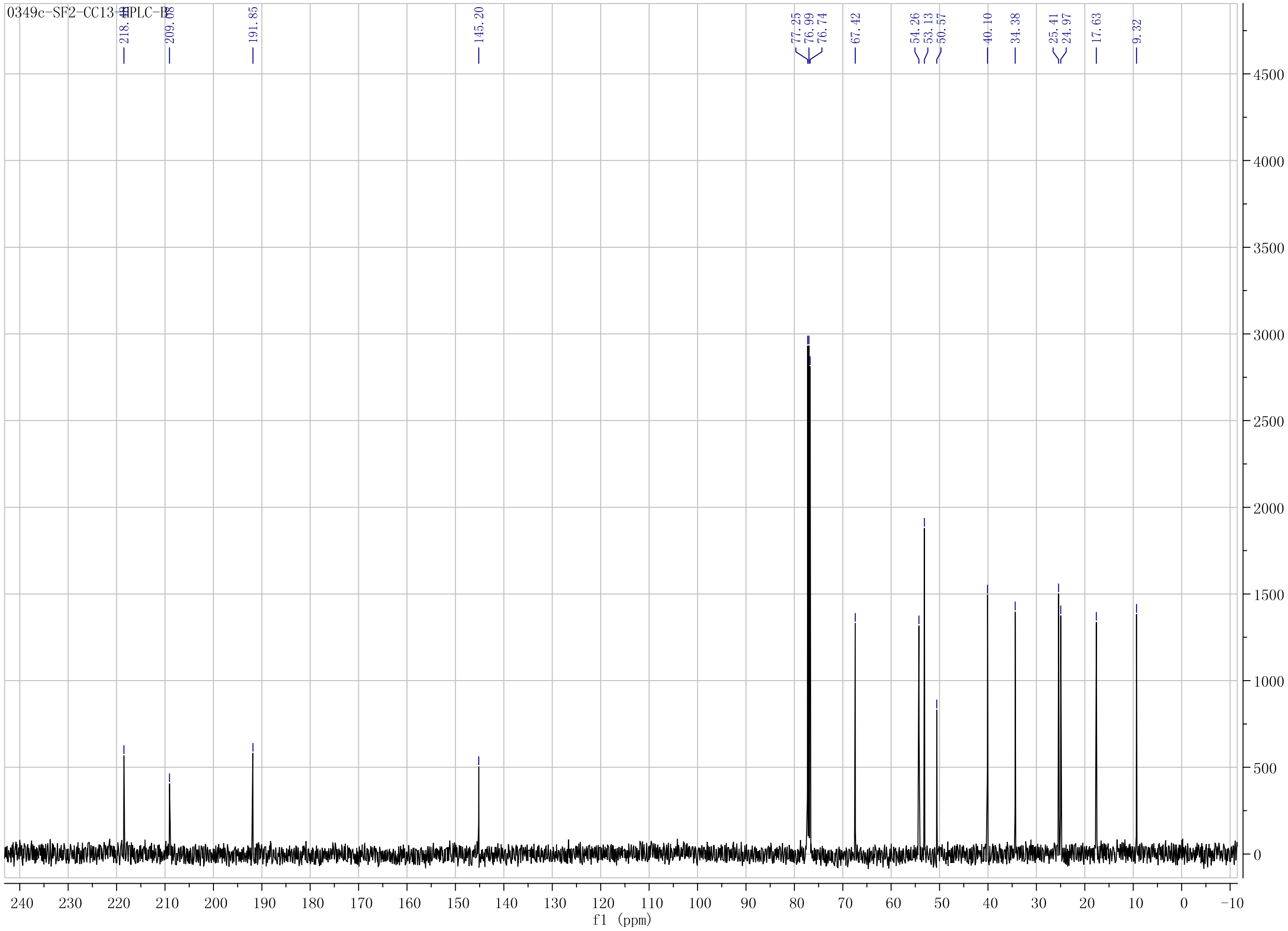

S17: ^{13}C NMR (125 MHz, $CDCl_3$) spectrum of chondrosterin C (**3**)

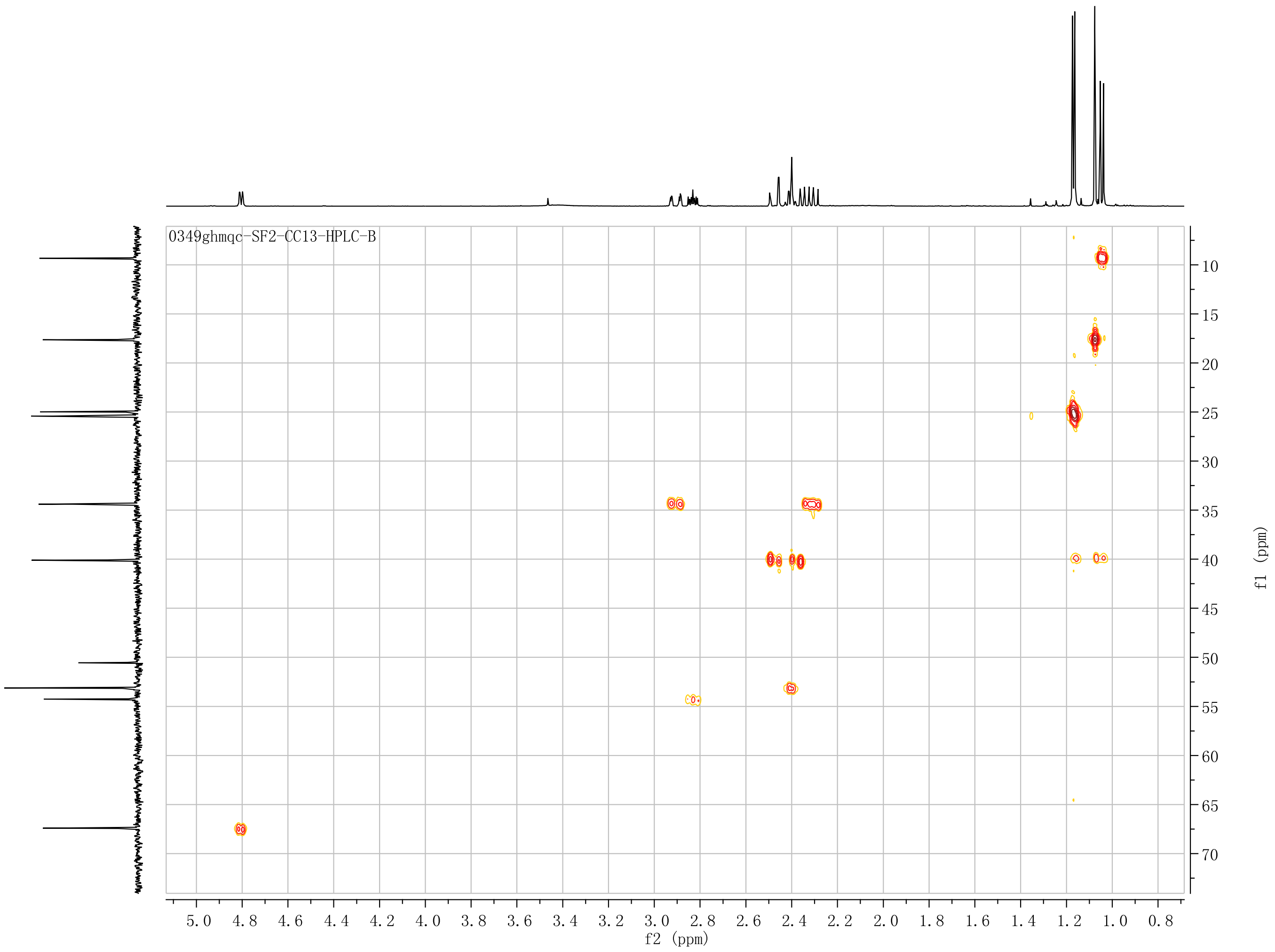

S18: gHMQC of chondrosterin C (**3**)

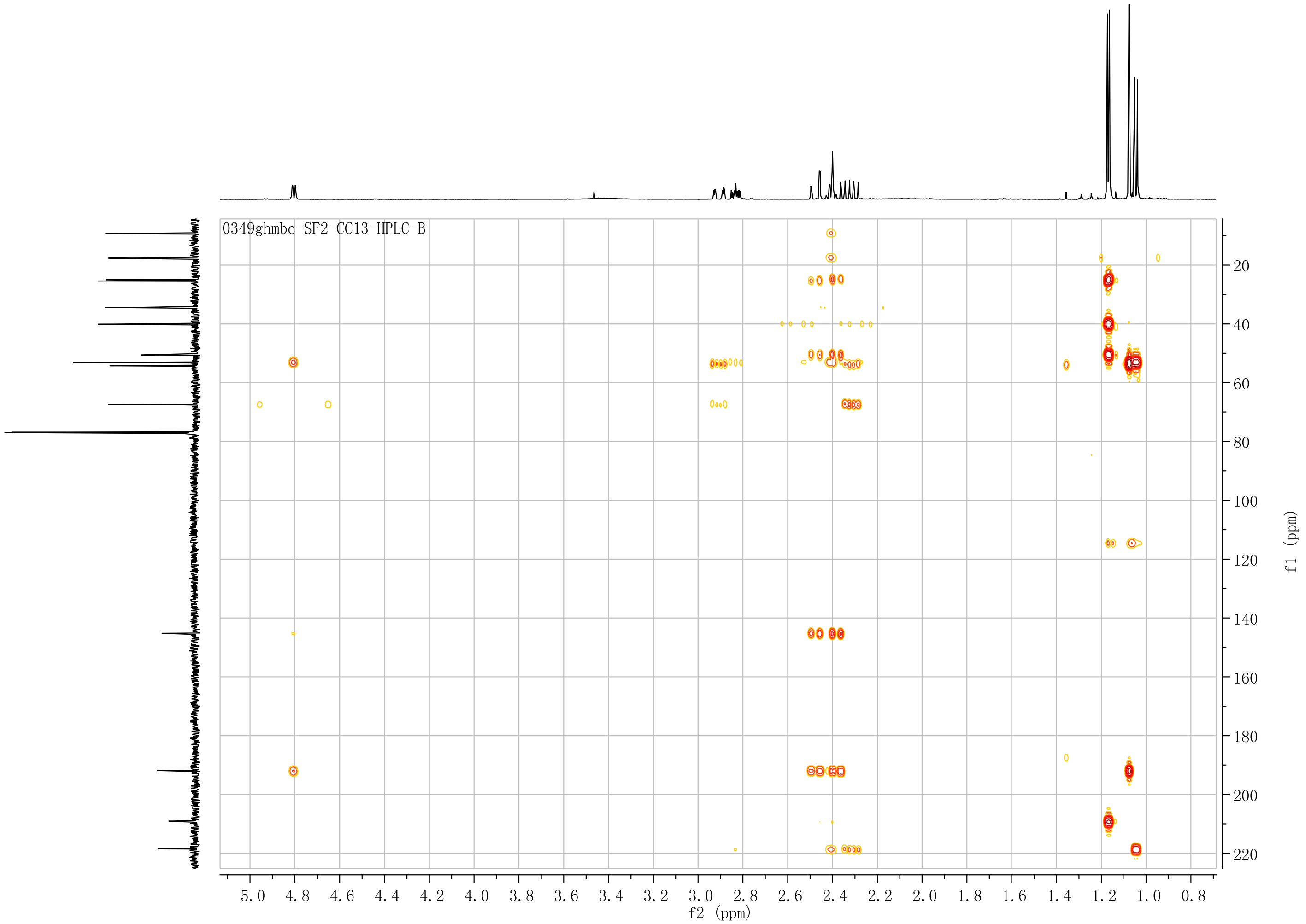

S19: gHMBC of chondrosterin C (**3**)

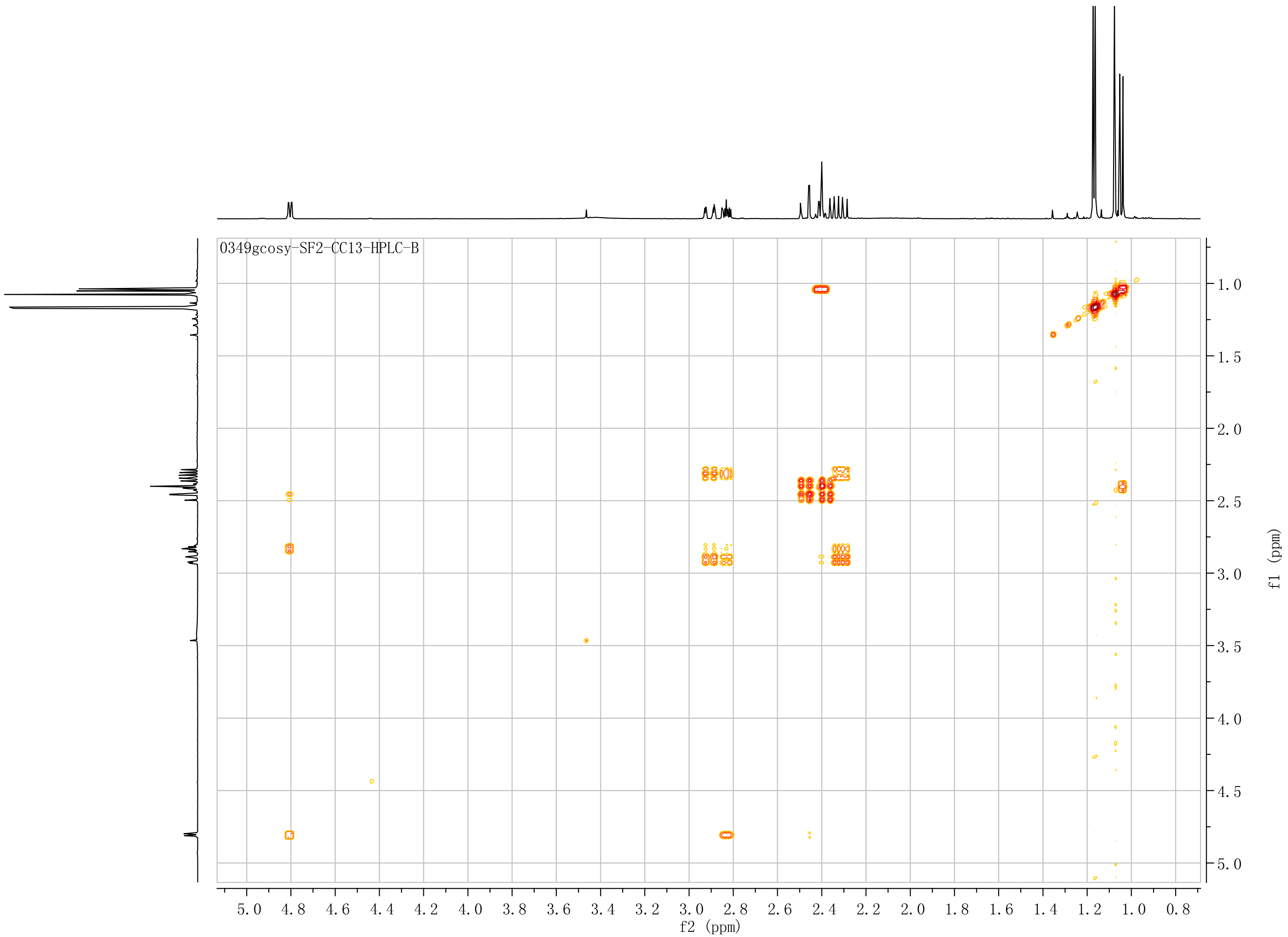

S20: ${}^{1}H$–${}^{1}H$ gCOSY of chondrosterin C (**3**)

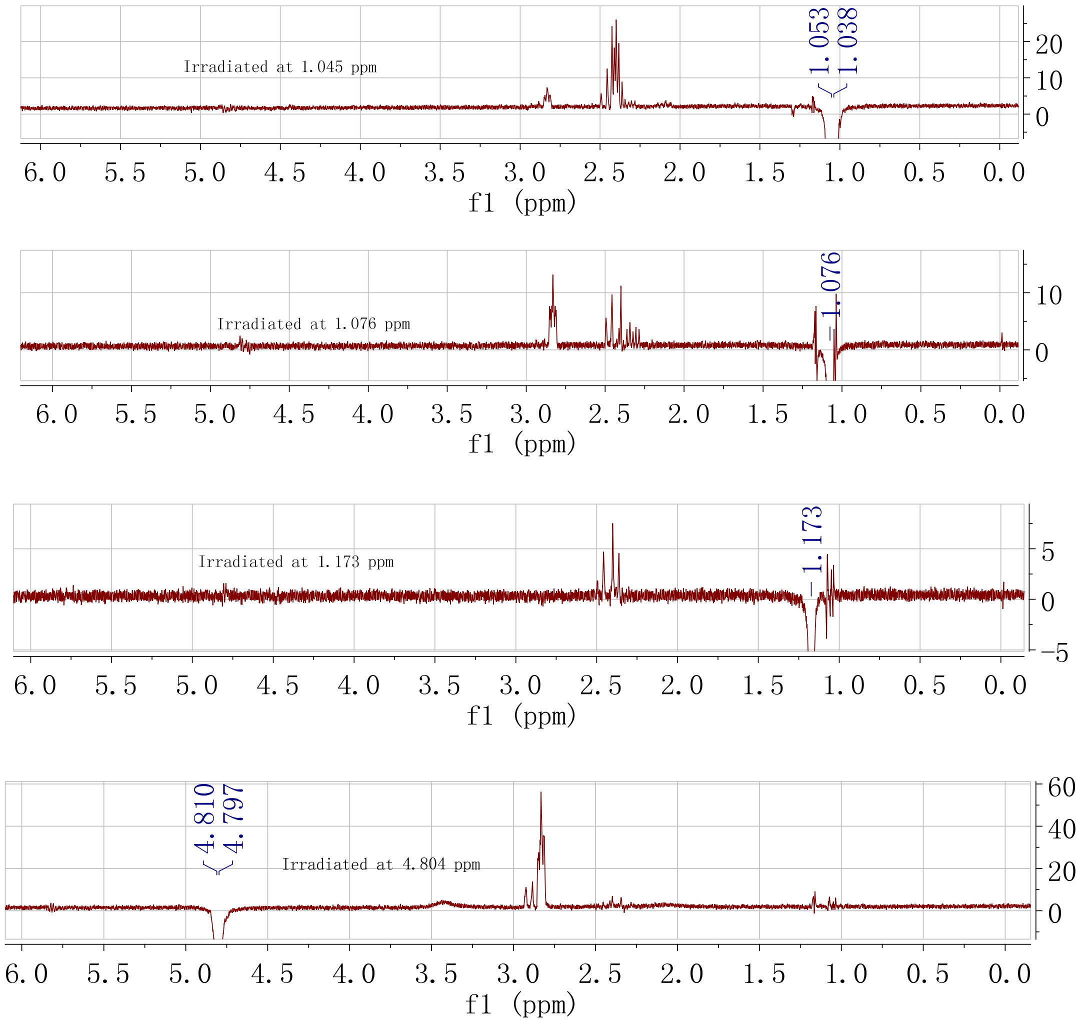

S21: NOE of chondrosterin C (**3**)

Full ms [242.100 - 255.700] - Range: 242.100 - 255.700

Scan No. 5 of 18

Scan #: 5

RT: 0.24

Data points: 1

Mass	Relative Intensity	Theoretical Mass	Delta[ppm]	Delta[mmu]	RDB	Composition
246.1255	100.0	246.1250	1.9	0.5	7.0	C_{15} H_{18} O_3

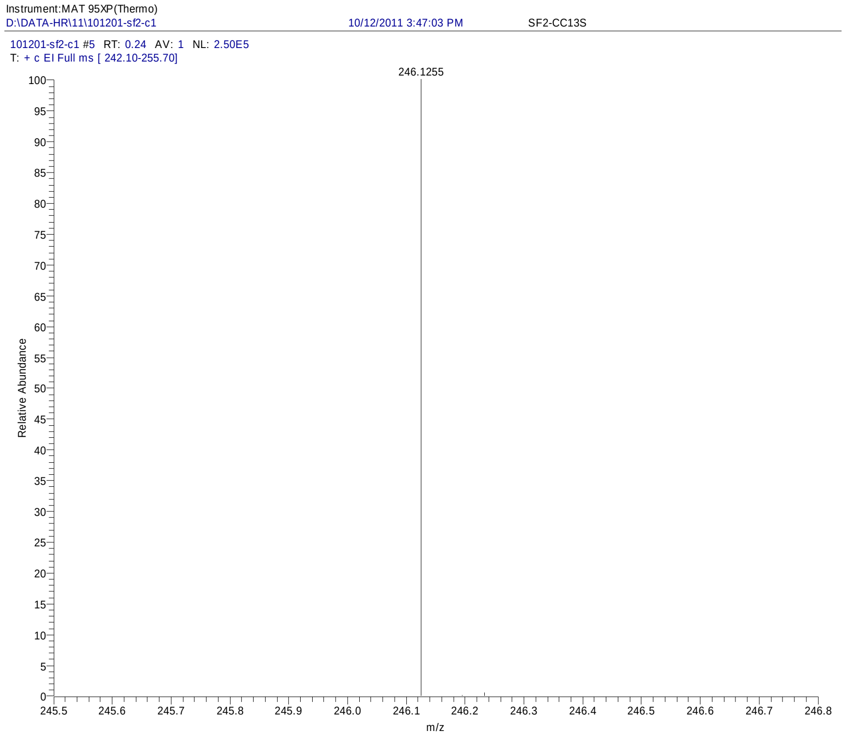

S22: HREIMS of chondrosterin D (**4**)

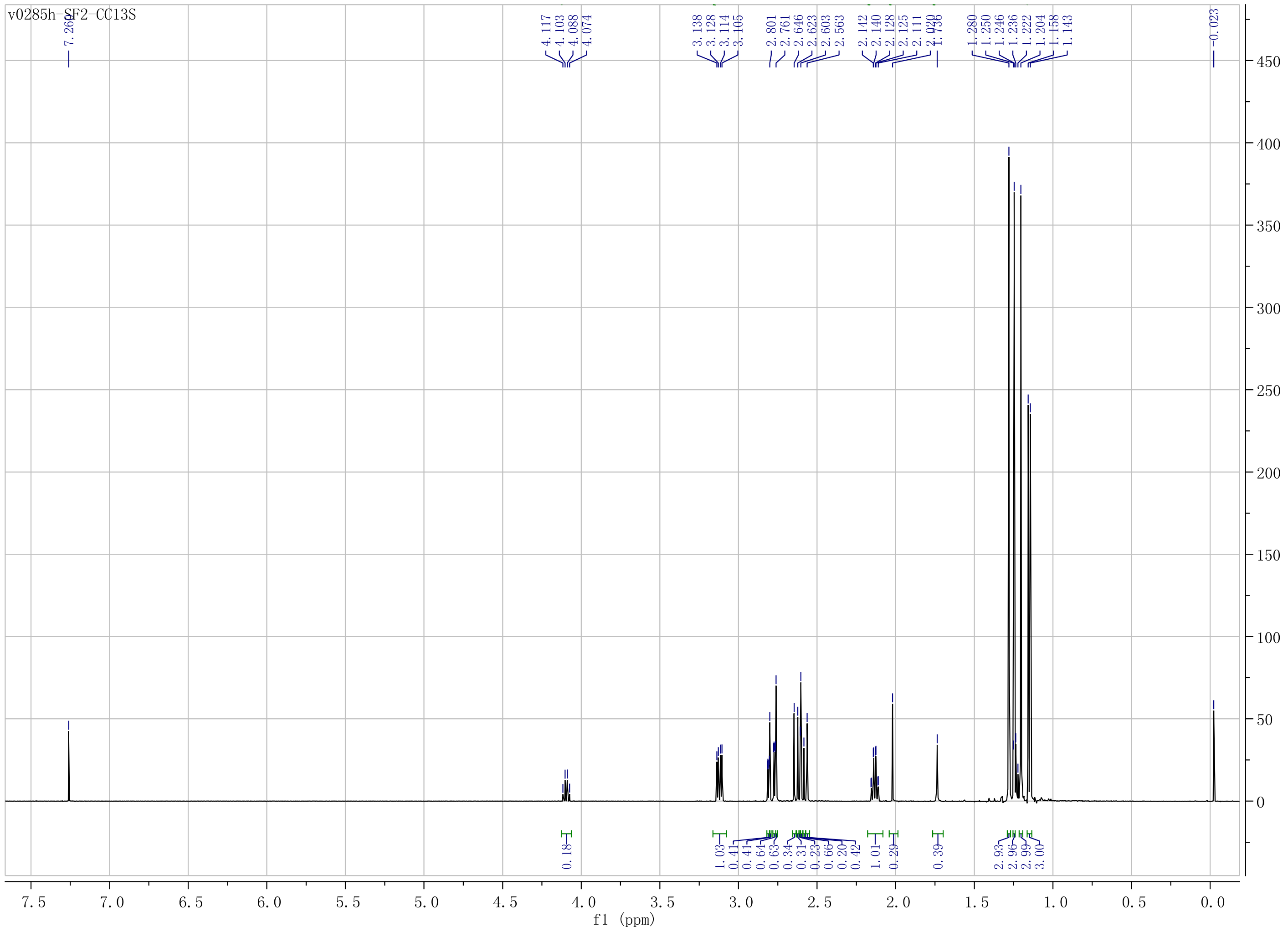

S23: ^{1}H NMR (500 MHz, $CDCl_3$) spectrum of chondrosterin D (**4**)

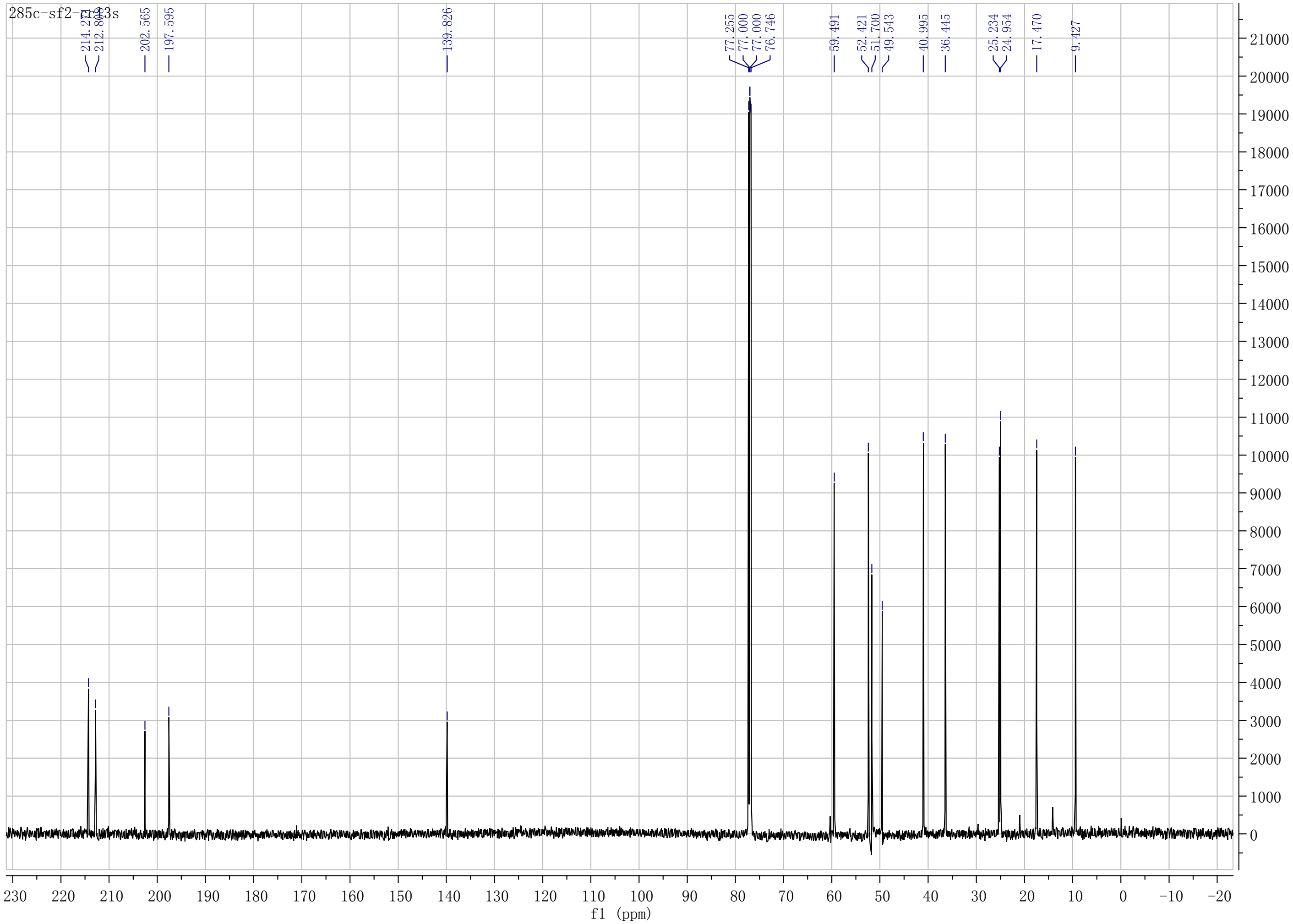

S24: ^{13}C NMR (125 MHz, $CDCl_3$) spectrum of chondrosterin D (**4**)

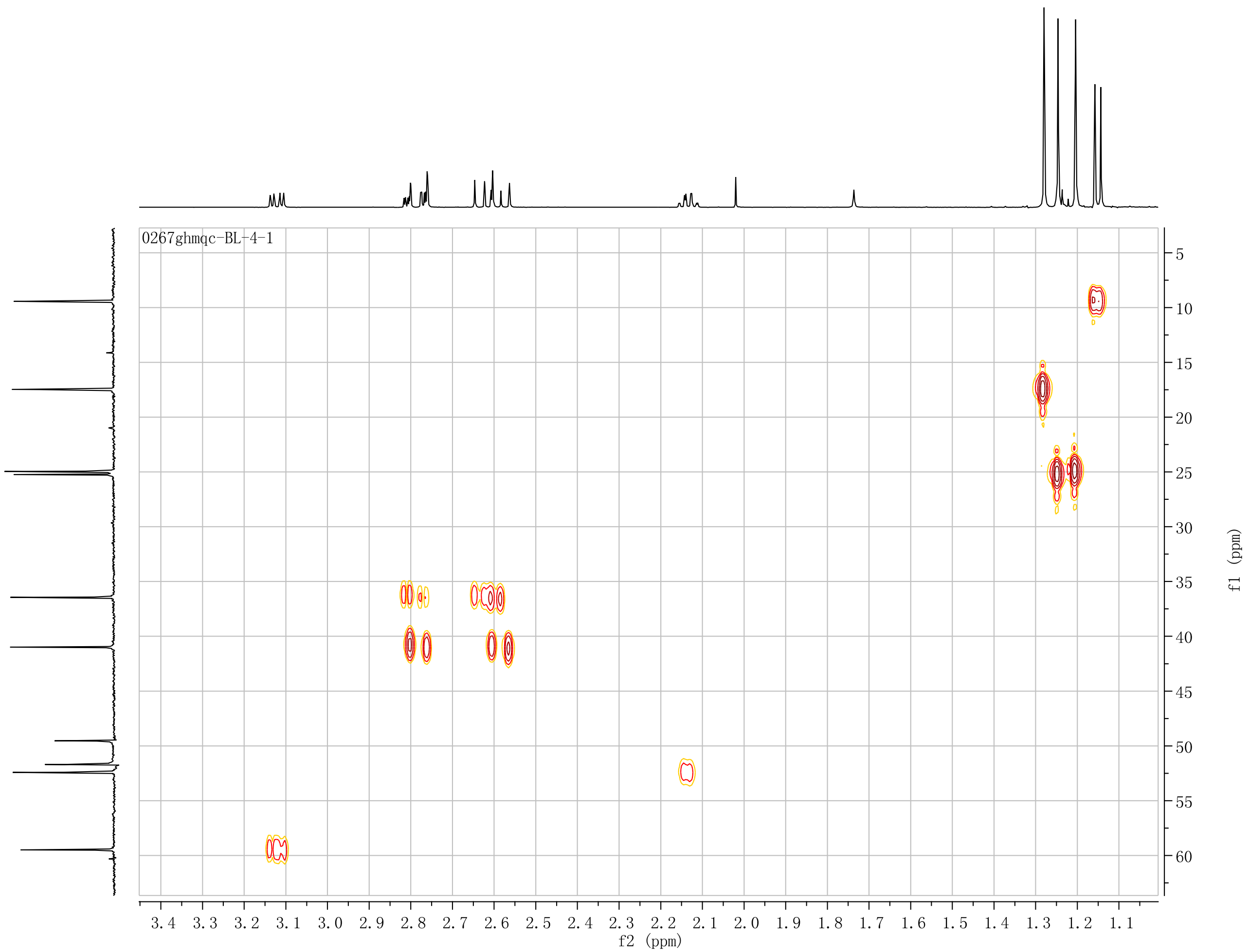

S25: gHMQC of chondrosterin D (**4**)

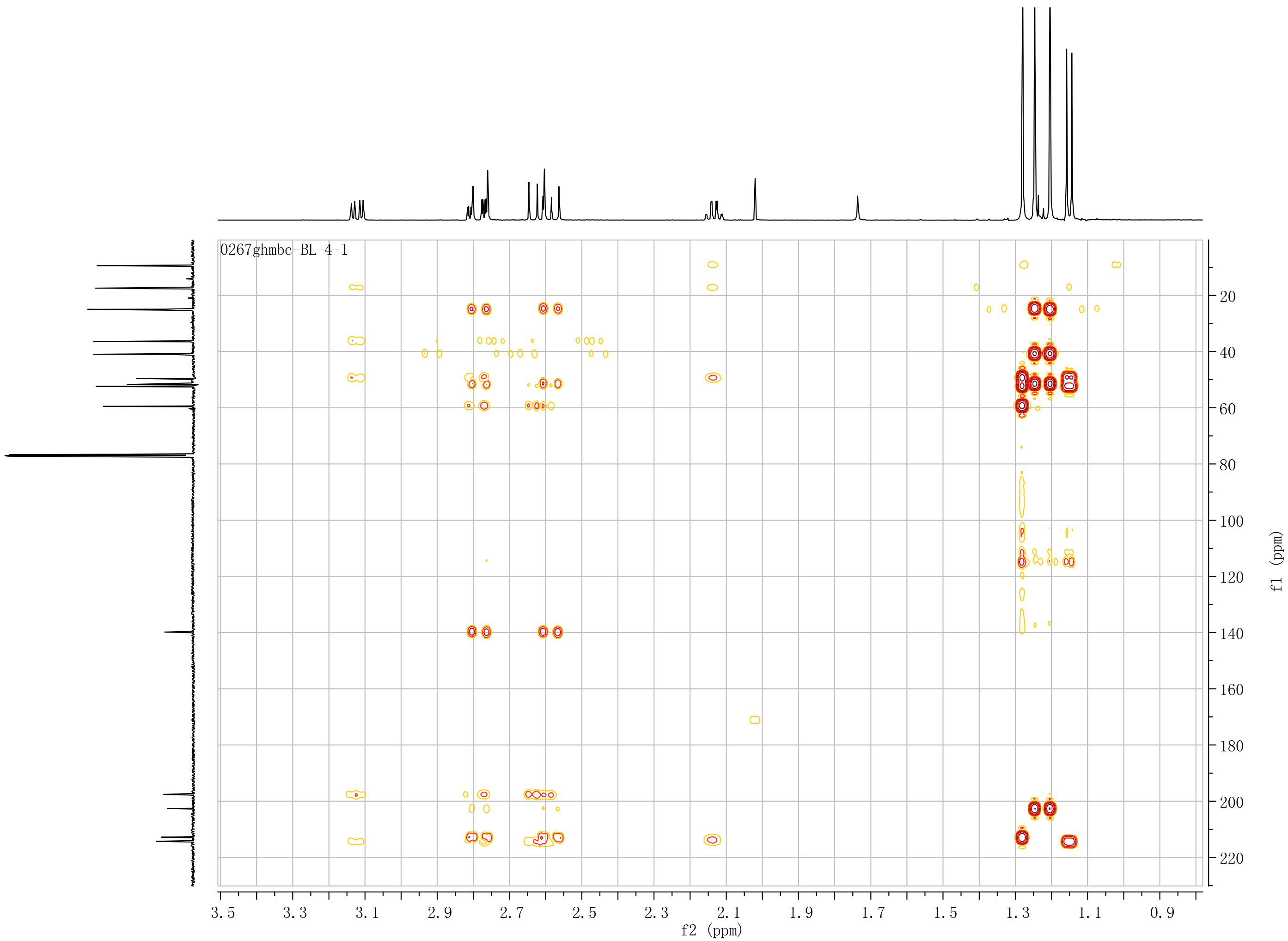

S26: gHMBC of chondrosterin D (**4**)

Full ms [227.500 - 246.500] - Range: 234.000 - 234.500

Scan No. 12 of 14

Scan #: 12

RT: 0.46

Data points: 1

Mass	Relative Intensity	Theoretical Mass	Delta[ppm]	Delta[mmu]	RDB	Composition
234.1613	65.5	234.1614	-0.4	-0.1	5.0	C_{15} H_{22} O_2

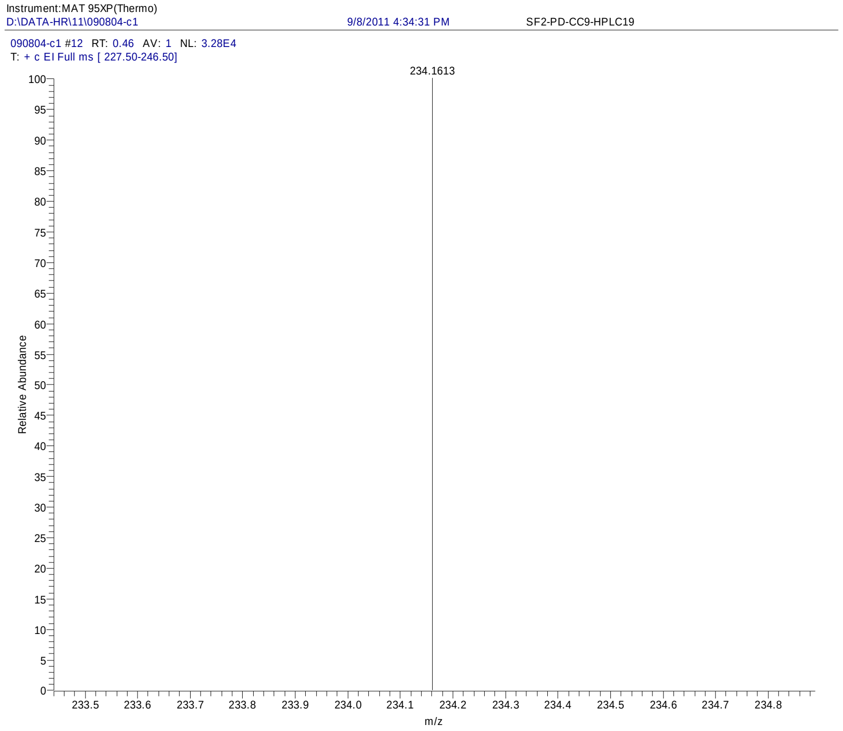

S27: HREIMS of chondrosterin E (**5**)

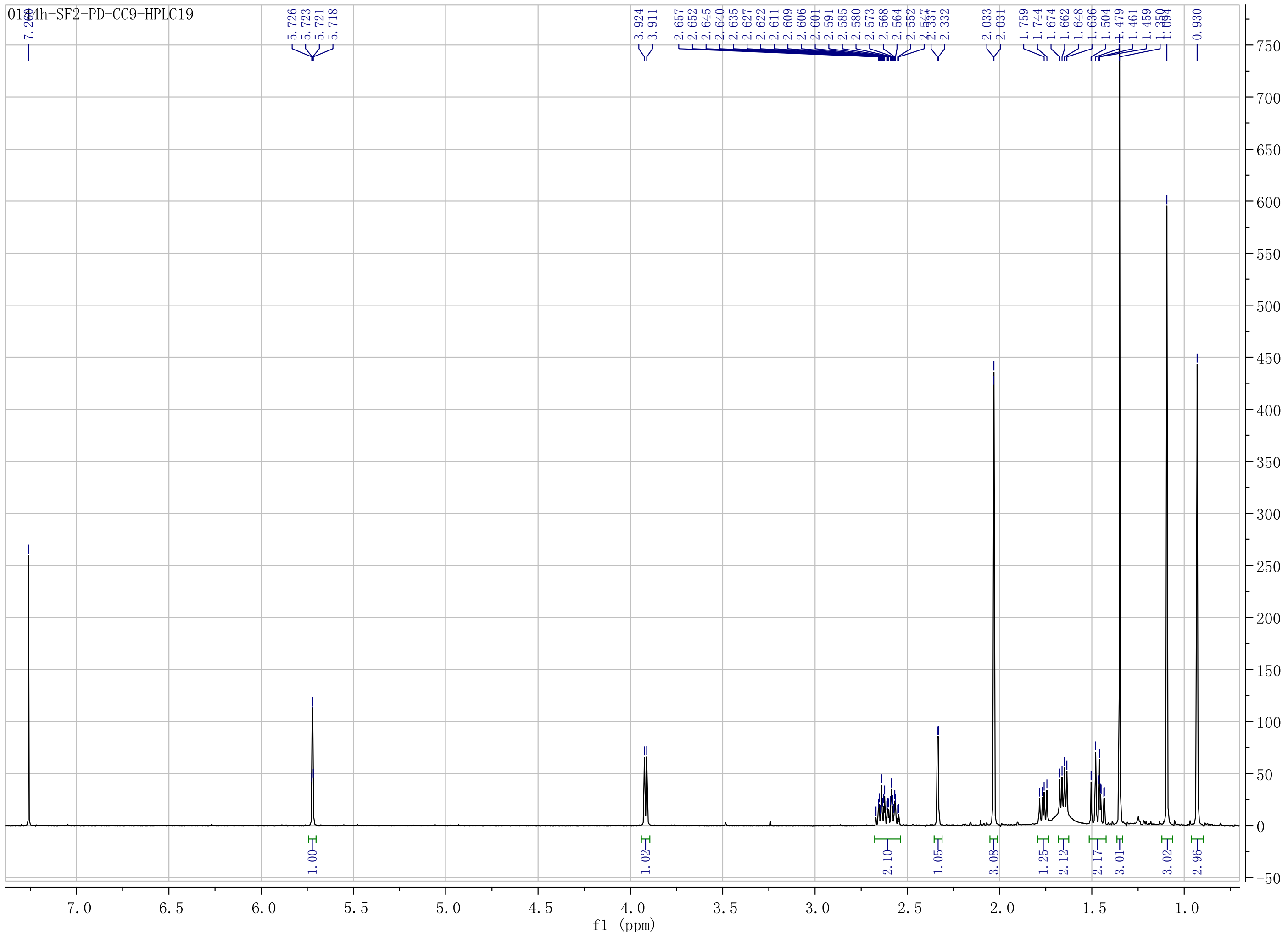

S28: ^{1}H NMR (500 MHz, $CDCl_3$) spectrum of chondrosterin E (**5**)

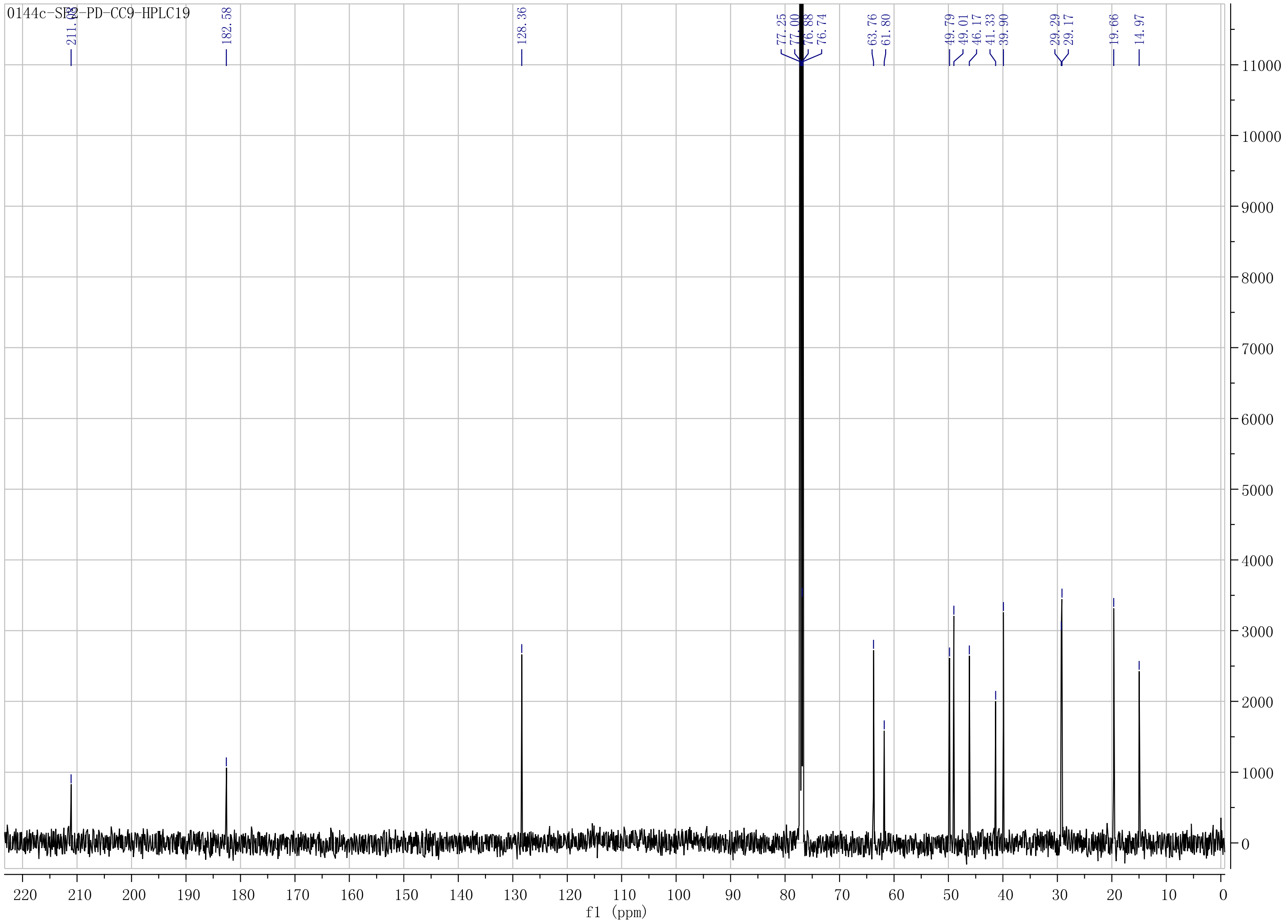

S29: ^{13}C NMR (125 MHz, $CDCl_3$) spectrum of chondrosterin E (**5**)

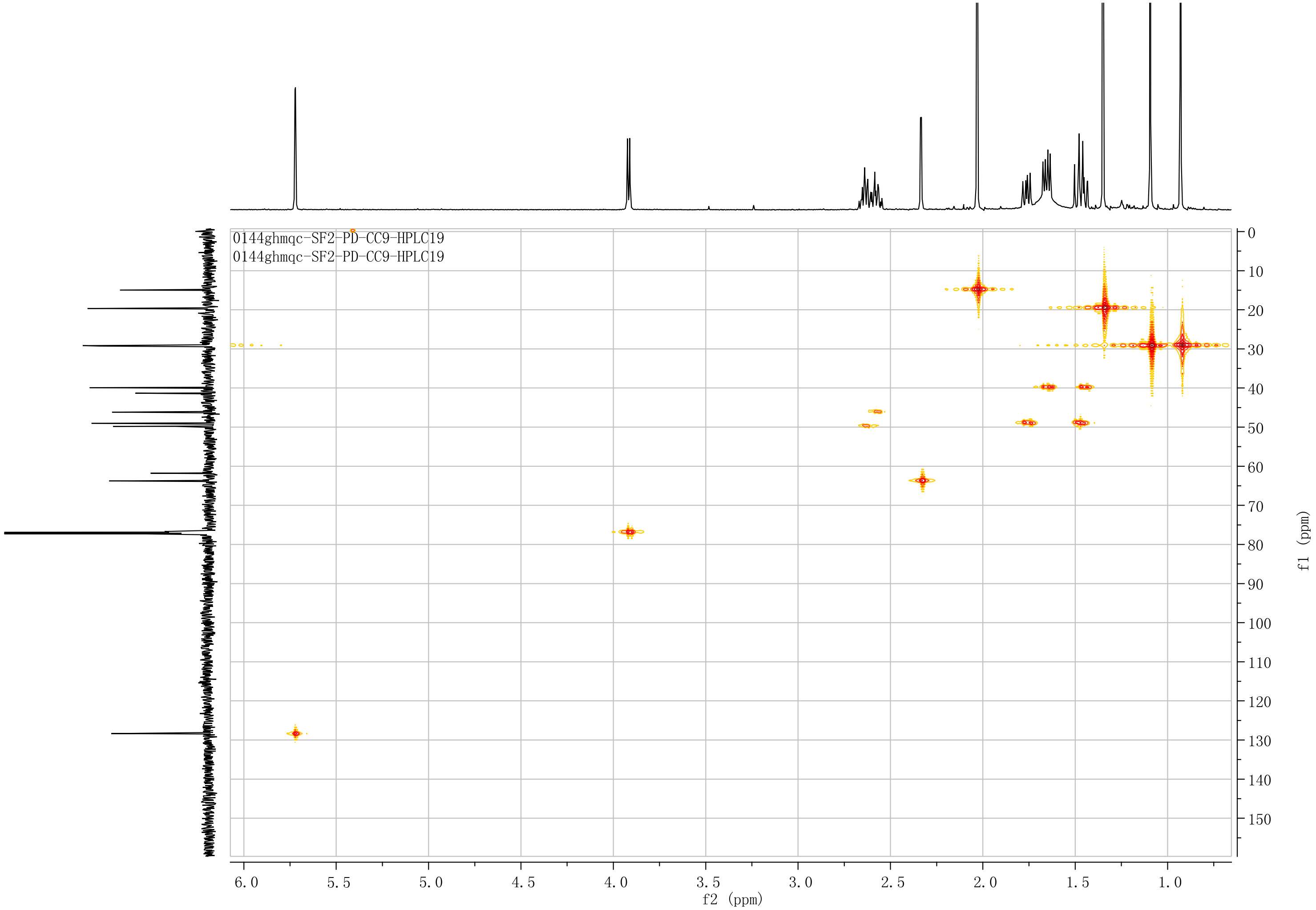

S30: gHMQC of chondrosterin E (**5**)

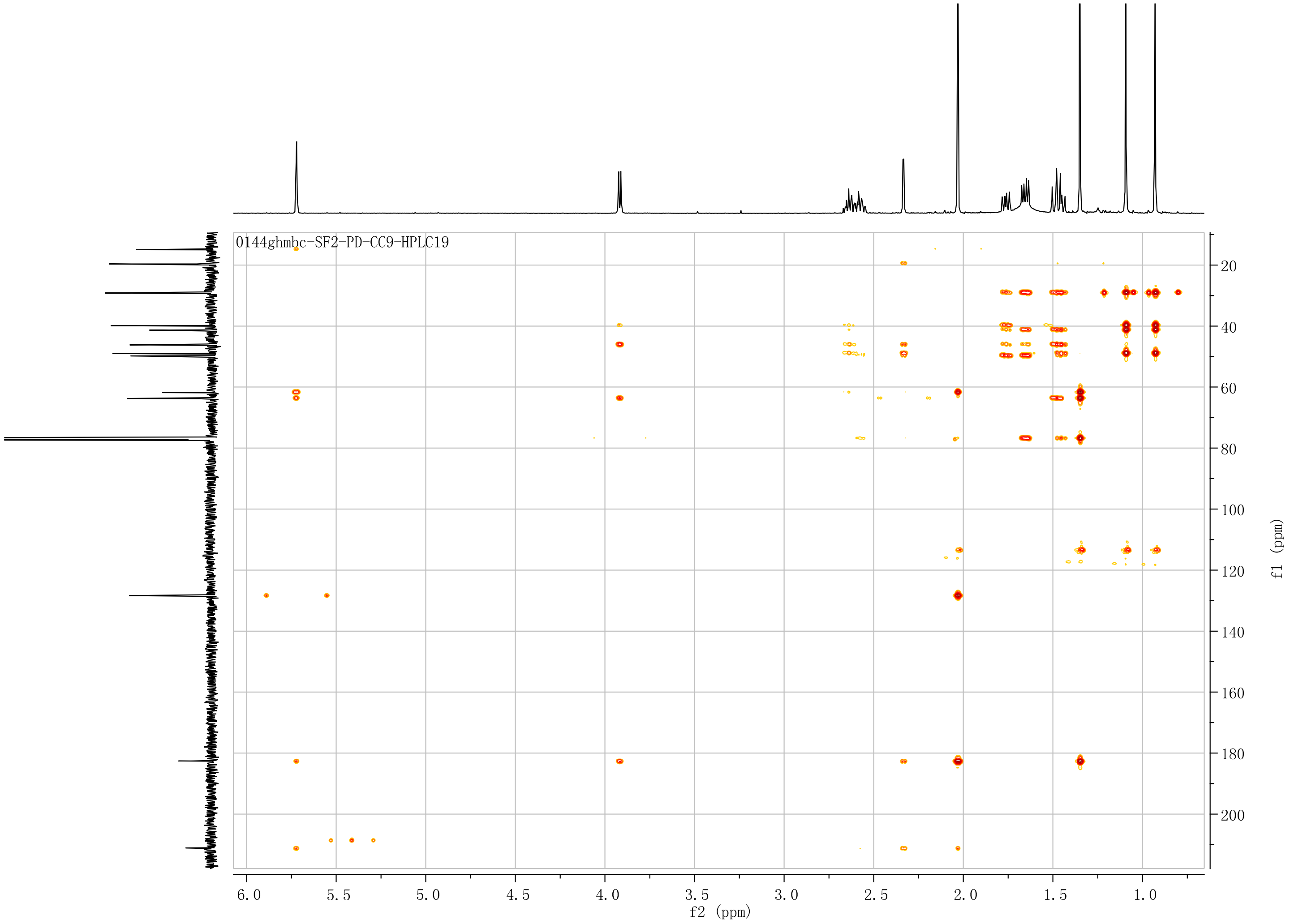

S31: gHMBC of chondrosterin E (**5**)

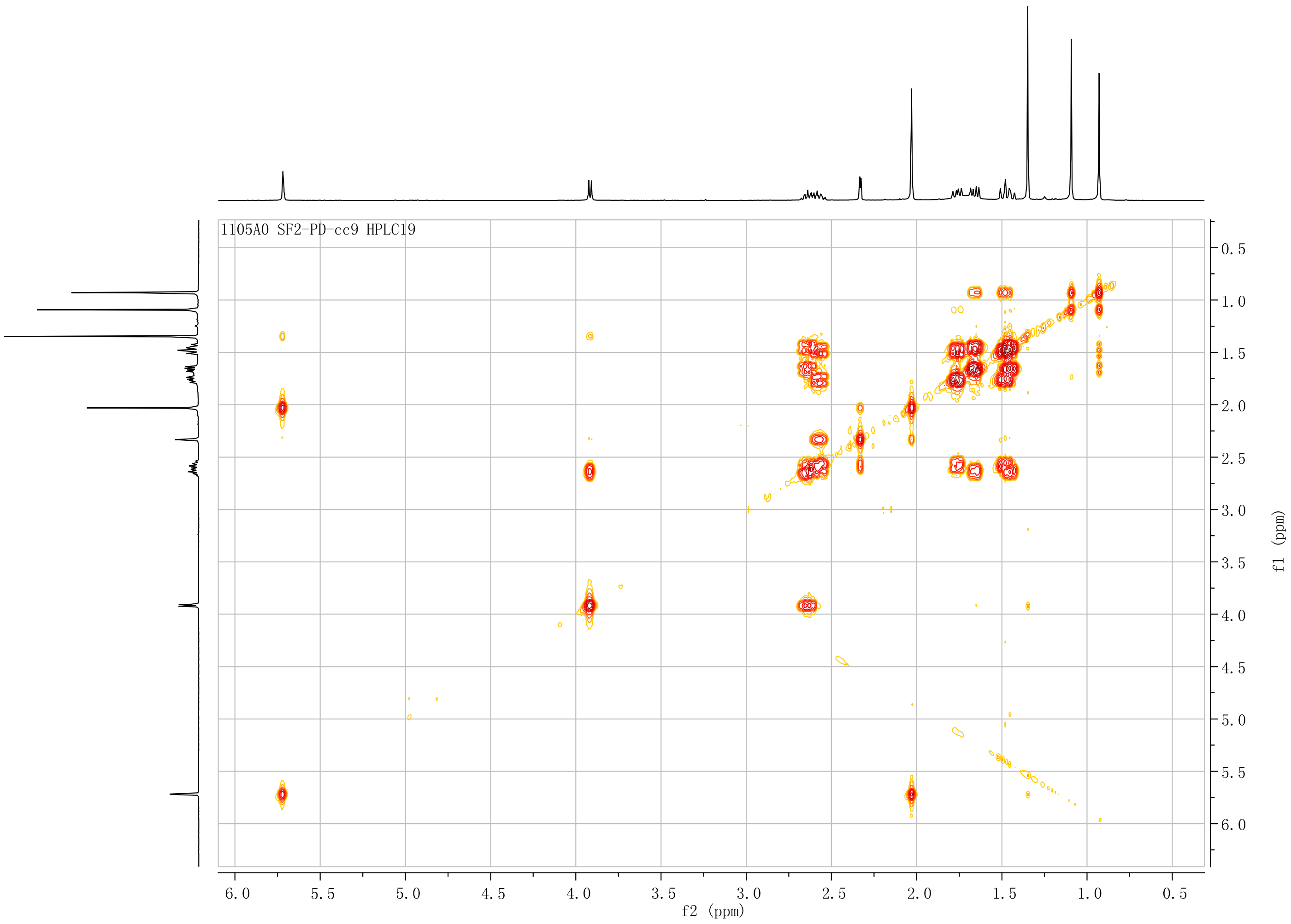

S32: ^{1}H–^{1}H gCOSY of chondrosterin E (**5**)

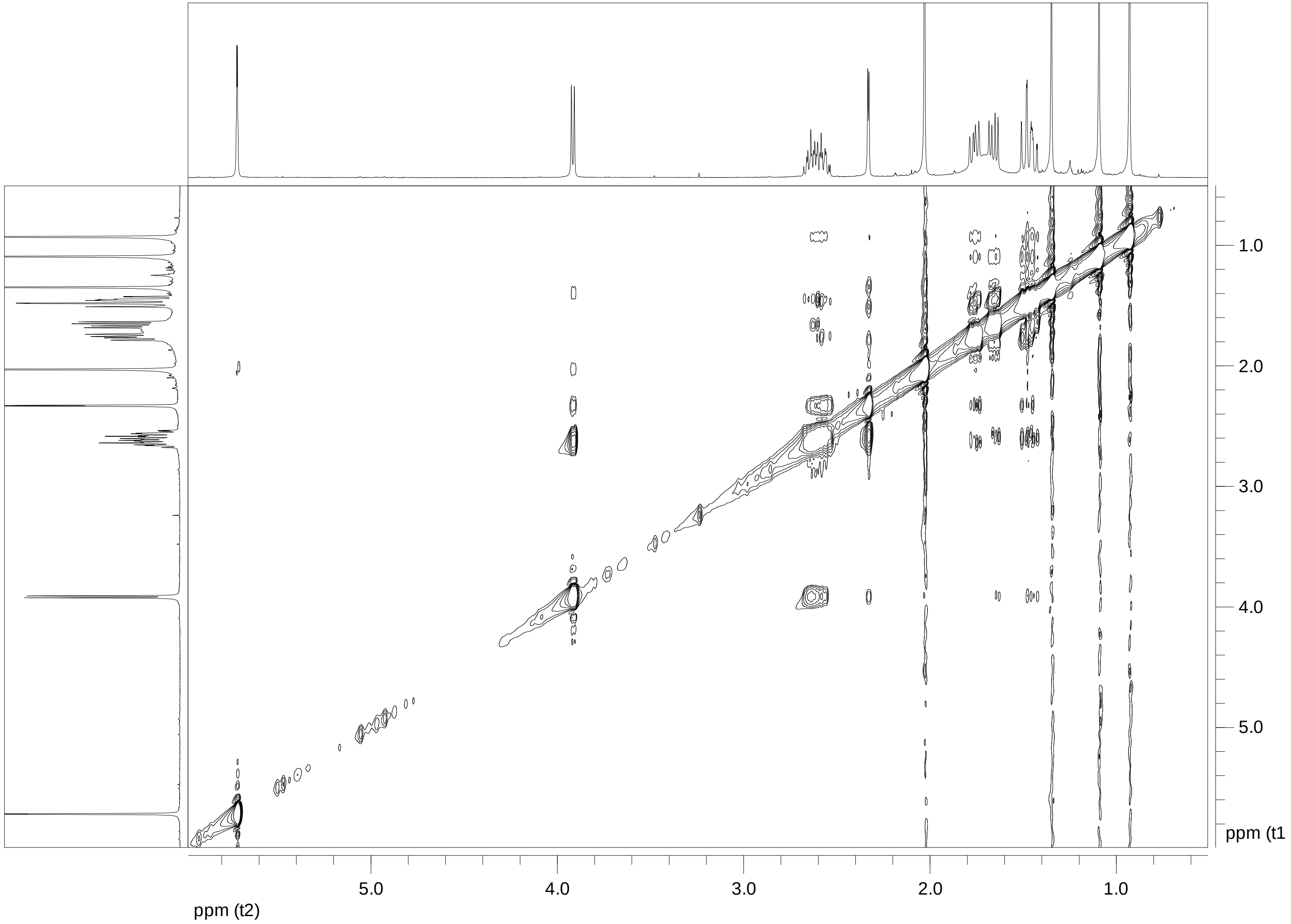

S33: ROESY of chondrosterin E (**5**)
